# Predicting future grizzly bear habitat use in the Bitterroot Ecosystem under recolonization and reintroduction scenarios

**DOI:** 10.1371/journal.pone.0308043

**Published:** 2024-09-04

**Authors:** Sarah N. Sells, Cecily M. Costello

**Affiliations:** 1 U.S. Geological Survey, Montana Cooperative Wildlife Research Unit, Wildlife Biology Program, Ecology and Evolution Program, University of Montana, Missoula, Montana, United States of America; 2 Montana Fish, Wildlife and Parks, Kalispell, Montana, United States of America; Smithsonian Conservation Biology Institute, UNITED STATES OF AMERICA

## Abstract

Many conservation actions must be implemented with limited data. This is especially true when planning recovery efforts for extirpated populations, such as grizzly bears (*Ursus arctos*) within the Bitterroot Ecosystem (BE), where strategies for reestablishing a resident population are being evaluated. Here, we applied individual-based movement models developed for a nearby grizzly bear population to predict habitat use in and near the BE, under scenarios of natural recolonization, reintroduction, and a combination. All simulations predicted that habitat use by grizzly bears would be higher in the northern half of the study area. Under the natural recolonization scenario, use was concentrated in Montana, but became more uniform across the northern BE in Idaho over time. Use was more concentrated in east-central Idaho under the reintroduction scenario. Assuming that natural recolonization continues even if bears are reintroduced, use remained widespread across the northern half of the BE and surrounding areas. Predicted habitat maps for the natural recolonization scenario aligned well with outlier and GPS collar data available for grizzly bears in the study area, with Spearman rank correlations of ≥0.93 and mean class values of ≥9.1 (where class 10 was the highest relative predicted use; each class 1–10 represented 10% of the landscape). In total, 52.4% of outlier locations and 79% of GPS collar locations were in class 10 in our predicted habitat maps for natural recolonization. Simulated grizzly bears selected habitats over a much larger landscape than the BE itself under all scenarios, including multiple-use and private lands, similar to existing populations that have expanded beyond recovery zones. This highlights the importance of recognizing and planning for the role of private lands in recovery efforts, including understanding resources needed to prevent and respond to human-grizzly bear conflict and maintain public acceptance of grizzly bears over a large landscape.

## Introduction

Conservation decisions often must be made with limited data. For example, planning recovery of extirpated populations into their former ranges often means planning without many, if any, locally gathered and temporally relevant data on how a species will use proposed habitats. In these situations, conservationists often rely on extrapolation of research conducted elsewhere (e.g., [1–3]). However, accuracy of extrapolated models is often unknown, as models are rarely tested for their transferability beyond the original scales at which the models were developed [4].

**Fig 1:**
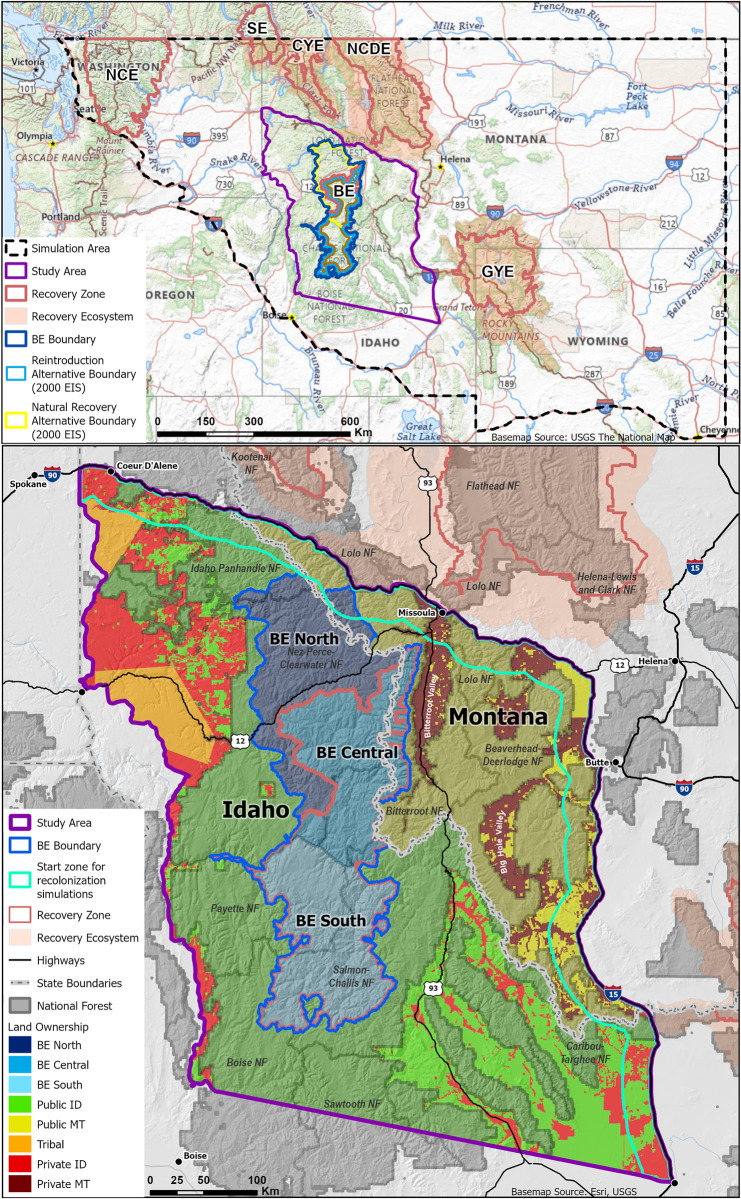
Study area. Our study area encompassed central Idaho and west-central Montana surrounding the reintroduction (Alternative 1) and natural recovery (Alternative 2) recovery zones identified in the 2000 Final EIS (USFWS 2000), together identified as the Bitterroot Ecosystem (BE) for this study (top panel). We subset the BE boundary into 3 regions: BE North, Central, and South. The BE overlaps various national forests, and nearly all of BE Central and South are designated wilderness (bottom panel; see also Figs A1 –A3 in S1 Appendix).

Conservation of grizzly bears (*Ursus arctos*) provides an excellent case study of the dilemma of how best to plan conservation efforts for populations with limited data. Grizzly bears were extirpated across 98% of their former range in the contiguous United States in recent centuries. Following listing as threatened under the Endangered Species Act (ESA) in 1975 and concerted conservation efforts over subsequent decades, population sizes have increased in four recovery areas: the Selkirk Ecosystem (SE), Cabinet-Yaak Ecosystem (CYE), Northern Continental Divide Ecosystem (NCDE), and Greater Yellowstone Ecosystem (GYE; Fig 1). The species now occupies 6% of their historical range in the contiguous U.S. [5]. Two other recovery areas were identified where remnant populations were known or thought to exist in the Bitterroot Ecosystem (BE) and North Cascades Ecosystem (NCE), but later evidence indicated these populations had also become extirpated.

Grizzly bears were once widespread in the BE region of east-central Idaho and western Montana, but the last verified death in the historical population occurred in 1932, and the last verified sign in the 20^th^ century was documented in 1946 [6]. The BE region includes one of the largest areas of public lands in the contiguous US, encompassing national forest lands with multiple designated wilderness areas. Following ESA protections in 1975, the Grizzly Bear Recovery Plan [7] called for research into the BE’s potential to support grizzly bears. Studies have indicated that the BE could likely support 200–400 individuals [8]. Reestablishing the population would contribute to long-term persistence of grizzly bears in the lower 48 states by improving the “3Rs” of resiliency, redundancy, and representation [9]. In 2000, upon completing an environmental impact statement (EIS), the US Fish and Wildlife Service (USFWS) published a record of decision and final rule with a preferred alternative for reestablishing a population in the BE through reintroduction as a non-essential experimental population under Section 10(j) of the ESA [10]. However, action on reintroduction was never taken, making natural recolonization the *de facto* process for recovery. Recently, the delay of agency actions to reestablish a BE population was challenged in court and the USFWS was ordered to prepare a supplemental EIS and, if warranted, a new record of decision and final rule [11].

Much has changed since completion of the 2000 EIS [10], including knowledge about grizzly bears and distributions of existing populations. Whereas the USFWS [10] then stated that “the likelihood of recovery of grizzly bears in the BE through natural recolonization appears remote because grizzly bears do not move far to colonize distant, disjunct areas,” recent data and analyses suggest that natural recolonization may be feasible. Since 2007, when a hunter mistakenly killed a large male grizzly in the BE [12], numerous observations and GPS-movements of grizzly bears have been documented in and near the BE. By 2022, the NCDE estimated occupied range had expanded to slightly overlap the recognized BE Recovery Zone [13]. Multiple long-range dispersal movements have been identified using DNA-based parentage analyses [13–15]. Studies examining recent expansion of grizzly bear populations have demonstrated the capacity and mechanisms by which humans and grizzly bears can share more human-dominated landscapes, to a larger degree than anticipated in the 2000 EIS [16, 17]. Finally, the successful augmentation of the population in the Cabinet Mountains of Montana [15, 18] and successful reintroductions of brown bears into the Italian Alps [19] have provided information relevant to reintroduction or augmentation options for the BE.

Despite many recent updates to knowledge of grizzly bear ecology, recent research that directly informs conservation planning within the BE is lacking. To address this gap, our research team recently developed highly predictive movement models for individual grizzly bears monitored with GPS collars in the NCDE [20], verified the models’ transferability to the SE, CYE, and GYE [21], and used them to predict connectivity pathways between populations, including the BE [22]. To assist with recovery planning for the BE, our goal was to use these models to predict habitat use within the BE region under scenarios of natural recolonization and reintroduction. Given that naturally recolonizing or reintroduced carnivores, especially habitat generalists like grizzly bears, often select habitats over a broader landscape than designated core areas [23, 24], a primary aim was to evaluate the relative distribution of selected habitat across recovery area boundaries, ownerships, and jurisdictions. Our mechanistic and landscape-wide approach supplements earlier studies that evaluated grizzly bear habitat in the BE region, based on natural and anthropogenic factors [25–30].

## Methods

### Study area

Our 65,084 km^2^ study area (Fig 1) included central Idaho and west-central Montana surrounding the reintroduction (Alternative 1) and natural (Alternative 2) recovery zones presented in the 2000 EIS [10], together identified as the BE for this study (22,244 km^2^). Within the BE, we defined BE North as the extent of the natural recovery alternative boundary outside the reintroduction alternative boundary. BE Central was the area of overlap for the reintroduction and natural recovery alternative boundaries. BE South was the remaining southern portion of the reintroduction boundary.

Our study area was bounded on the west by U.S. Highway 95 and Idaho Highway 55 (south to Banks); on the north by Interstate-90 (I-90); and on the east by Interstate 15 (I-15; south to Idaho Falls). This area included the non-essential experimental population boundary, i.e., 10(j) area, identified in the 2000 EIS [10], but was extended eastward and southward to allow us to simulate natural recolonization. To accommodate simulations that sometimes extended outside of the study area, the study area was situated within a wider simulation area where covariate data were developed for our studies [20], including portions of Washington, Oregon, Idaho, Montana, and Wyoming.

The study area ranged 242–3684 m in elevation. The northwest portion of the study area, including BE North, was mountainous and rugged, with extensive forests [31]. The eastern third of the study area contained grass- or shrub-filled intermontane valleys, foothills with shrubs, grasses, or forest, and high mountains with forests and some high alpine zones. The remainder of the study area, including BE Central and BE South, was characterized by partially glaciated, granitic, mountainous terrain. Compared to BE North, BE South was warmer and drier with more open forests. Major features of the BE included the Lochsa, Selway, and Clearwater Canyons with cold, fast rivers and a warmer, drier climate in the canyon bottoms.

The study area comprised public, tribal, and private lands (Fig 1). The BE comprised 19% of the total study area and was >99% public land, including portions of numerous national forests (NF, including in BE North), wilderness areas (including nearly all of BE Central and BE South, together totaling >15,000 km^2^), and mountain ranges (Figs A1 –A3 in S1 Appendix). Ownership around the BE included public lands in Idaho (43%) and Montana (18%) primarily managed by the US Forest Service, including additional wilderness areas. Tribal lands comprised 3% of the study area, with private lands comprising the remainder of the study area in Idaho (10%) and Montana (5%). Key areas of private lands included the Bitterroot Valley to the northeast of the BE, the Big Hole Valley to the east of the BE, and various private lands around the Deerlodge NF in the northeastern corner of the study area. Forestry, ranching, agriculture, and recreation were major land uses. Most towns in the interior of the study area were small (<3,000 people), but several cities (>30,000 people) were present near the periphery of the study area, including Lewiston, Coeur d’Alene, Missoula, Butte, and Idaho Falls.

### Simulation overview

**Fig 2:**
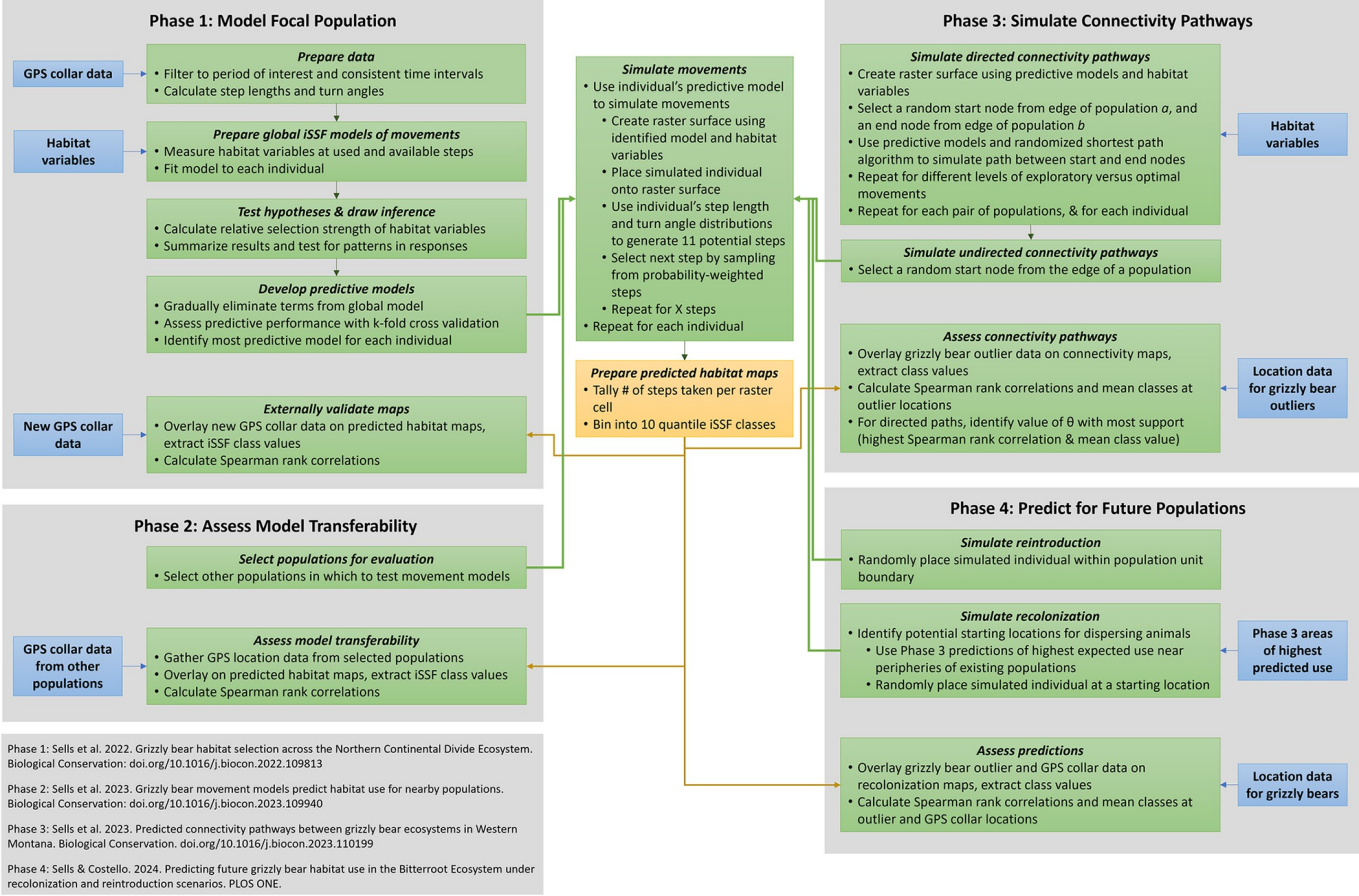
Phases of overall research. Our study represented the fourth phase of research aimed at providing science to help inform grizzly bear conservation. In the first phase, Sells et al. [20] developed and tested models for a focal population (the NCDE). In the second phase, Sells et al. [21] assessed model transferability by applying the models to nearby populations in the GYE, CYE, and SE. In the third phase, Sells et al. [22] applied the models to simulate connectivity pathways between populations in western Montana. In this present fourth phase, we applied the models to a recovery area without a current population, the BE.

We used the models developed in [20] for 65 NCDE grizzly bears (46 females and 19 males; S1 Appendix) to run simulations of grizzly bear movement and habitat use in the BE (Fig 2). Capture and handling of grizzly bears was conducted under permits issued by the USFWS for technical assistance pursuant to the 4(d) rule of the ESA. Protocols were approved by the Montana Fish, Wildlife and Parks Animal Care and Use Committee in writing [32]. As cooperating agencies for population monitoring activities, field site access was granted by the U.S. Forest Service, National Park Service, State of Montana, Blackfeet Nation, and Confederated Salish and Kootenai Tribes. Access to private lands were granted by individual landowners.

Each bear model consisted of an integrated step-selection function (iSSF), which compares covariates (300 x 300-m cell resolution) associated with animal locations and random locations accessible from each animal location [33]. The iSSF has exponential form, whereby *w*(x) = exp(xβ); *w*(x) is the iSSF score, x is a vector of habitat covariates, and β is the coefficient vector estimated via conditional logistic regression. Individual iSSFs were based on data collected at 3-hour intervals (+/- 45 min) from the 65 individuals bears monitored by GPS transmitters (Telonics, Mesa, Arizona, USA) for one or more years between 2004 and 2020 during the primary active season (May–Nov). Sells et al. [20] first created global iSSFs for each bear using package amt [34] in Program R [35]. Seven habitat covariates were included in the global iSSFs (S1 Appendix): the Normalized Difference Vegetation Index (NDVI, as an index to food abundance) during peak green-up (Jun 15 –Jul 15), terrain ruggedness, distance and density of forest edge, density of riparian areas, density of buildings, and distance to secure habitat (i.e., as defined by the US Fish and Wildlife Service: areas on public, state, and tribal lands *>*500 m from roads). Sells et al. [20] developed a final predictive iSSF for each bear by iteratively eliminating terms from the global iSSF to identify which model formulation maximized the cross-validation score for that individual. Some bears therefore had the global iSSF as their final model, and others had iSSFs with fewer variables.

As in previous publications [20–22] (Fig 2), we used each bear’s iSSF to create a conductance surface (300-m cell resolution) for that bear’s simulations in Program R [35]. We calculated conductance values across the simulation area as exp(β*xi*), where β was the coefficient vector of the estimated iSSF and *xi* the vector of habitat covariates of cell *i* [36]. We trimmed extremes using the 0.025 and 0.975 quantile values and normalized the remaining values to a 0–1 scale [37]. We then completed two sets of simulations.

### Natural recolonization simulations

In the first set of simulations, we simulated natural recolonization of the study area by dispersing grizzly bears from nearby populations. We defined a starting region for these recolonization simulations using a 15-km buffer south of I-90 and west of I-15 (Fig 1) to focus simulations on bears that had successfully passed this key anthropogenic landscape feature. Within this area, we generated 25,000 random locations ≥10 m apart. Using the predicted pathways based on undirected simulations starting from occupied range in Phase 3 [19], we then selected those random locations that overlapped cells in the highest 3 selection classes (8–10, representing the top 30% of predicted use from that study), omitting any locations within city boundaries. This yielded 7,971 start nodes for females and 5,686 for males (Figs A4 –A5 in S1 Appendix). We started each simulation within a randomly drawn start node. Following methods from the previous phases, for each sequential step, we generated 11 possible steps from the bear’s observed step length and turn angle distributions and sampled which step to go to from the probability-weighted steps (calculated as the iSSF value at the endpoint of each step divided by the sum of the 11 step values). We repeated this cycle of step selection for 20,000 steps to ensure opportunity for simulated bears to explore the study area. For each individual bear, we iterated this sequence 100 times for females and 242 times for males to yield approximately equal total iterations per sex (4,600 iterations for females and 4,598 iterations for males).

### Reintroduction simulations

In the second set of simulations, we simulated habitat use for a reintroduced population within the BE. In this round of simulations, we designated the BE as the start zone for simulated grizzly bears (Fig 1). Simulated bears were added to a start node drawn randomly from within this boundary and followed the same cycle as above for evaluating 11 possible steps, selecting a step, and moving there, for a total of 20,000 steps. We repeated the simulation iterations 100 times per female model and 242 times per male model.

### Predictive maps

To prepare results from simulations, we summed total steps per raster cell across iterations, for each sex and type of simulation (recolonization or reintroduction). We also combined results across sexes and for both simulation types to summarize overall results, representing full predictions of habitat use under a reintroduction scenario where natural recolonization occurs simultaneously. We next mapped relative predicted use of the study area by binning steps within the study area into 10 quantile classes of relative probability of use; lowest use was given class 1 and highest use class 10 [38].

To show how space use could shift during recovery, we prepared sequence maps. For each simulation type, we first summed and binned sequential sets of 25% of the total set of steps (i.e., Sequence 1 = steps 1–5,000, Sequence 2 = steps 5,001–10,000, … Sequence 4 = steps 15,001–20,000). The set of resulting sequence maps show the expected relative use of habitat by recolonizing or reintroduced grizzly bears from soonest (Sequence 1) to future (Sequence 4) time periods.

### Predictive capacity

We used available locations of grizzly bears to investigate predictive performance of our natural recolonization maps. Location data were from outlier observations (n = 63 since 2010) or collared grizzly bears that entered the study area (n = 6; Table A2 in S1 Appendix). State and federal agencies have documented grizzly bears in the study area in the form of generally isolated observations of presumably unmarked individuals verified with photo documentation of the bear(s) or their tracks. Observations were considered outliers if they occurred >7 km beyond the extent of the estimated occupied range in that year, and likely involved dispersing individuals. Although limited, these outlier and GPS collar data provide an initial evaluation of the predictive capacity of our recolonization maps. Therefore, we measured Spearman rank correlations between classes and numbers of outliers, the percentage of outliers in the top classes, and mean class at outlier locations. We repeated these measurements using the GPS collar data.

### Habitat summaries

Our final step was to summarize habitat characteristics. To reveal habitat grizzly bears could encounter, we plotted the median, 50% interquartile range, and 95% range for values of the 7 habitat covariates in each recovery ecosystem (averaging across sexes for sex-specific variables) and plotted results using ggplot2 [39]. The recovery ecosystems were the recovery zones plus their demographic monitoring areas (NCDE and GYE) or surrounding 10-mile (16.1 km) buffer zones (SE and CYE; Fig 1). For comparison, we calculated summaries for each of the BE subsections (North, Central, and South) for this analysis.

We next compared characteristics of habitat used by simulated grizzly bears. Although we consistently applied our models across all 4 phases of research (Fig 2), simulated bears encountered and responded accordingly to the unique habitat characteristics of each raster cell on the map. We thus measured mean values of the 7 habitat covariates within each class for the recolonization maps, reintroduction maps, and habitat maps from the NCDE (Phase 1 research, [20]), and SE/CYE and GYE (Phase 2 research, [21]). Results revealed habitat use in relation to habitat covariates (e.g., a positive relationship between habitat value and class value indicated that simulated bears selected for higher values of that habitat variable). Finally, we calculated the proportion of each land ownership type used by simulated grizzly bears by sex, simulation type, and class.

## Results

### Natural recolonization simulations

**Fig 3:**
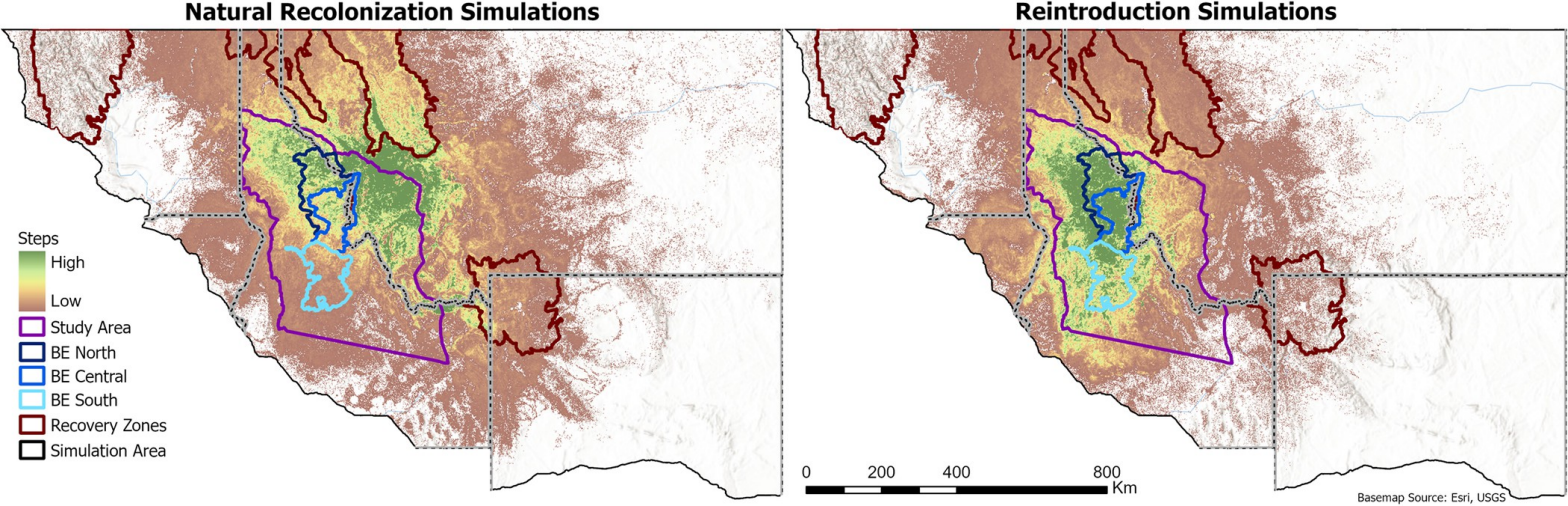
Maps of steps taken by simulated grizzly bears. Simulations were initiated on the north and eastern edges of the study area for the natural recolonization scenarios and within the BE for the reintroduction scenarios.

**Fig 4:**
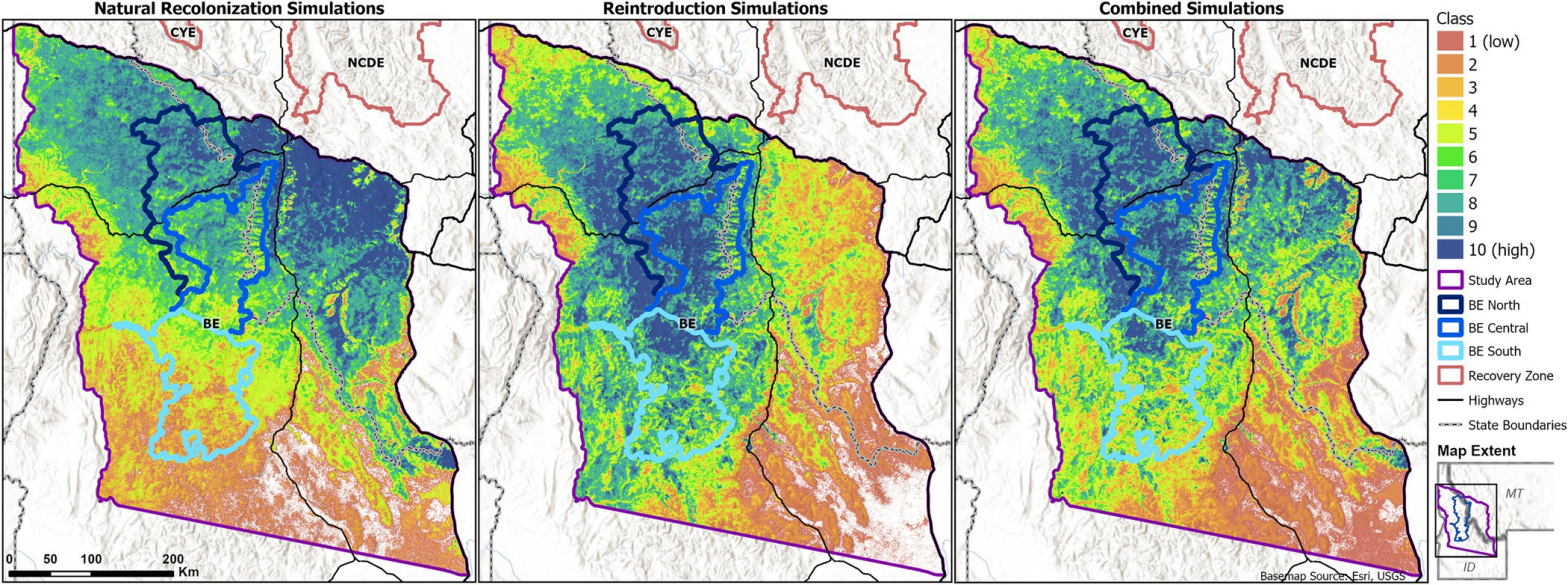
Maps of predicted habitat use for grizzly bears. Results are shown for the natural recolonization and reintroduction scenarios, and both scenarios combined. Classes represent the quantile-binned relative habitat use values (1 = low, 10 = high), as summarized within the study area based on the number of steps taken per 300- x 300-m grid cell (Fig 3).

**Fig 5:**
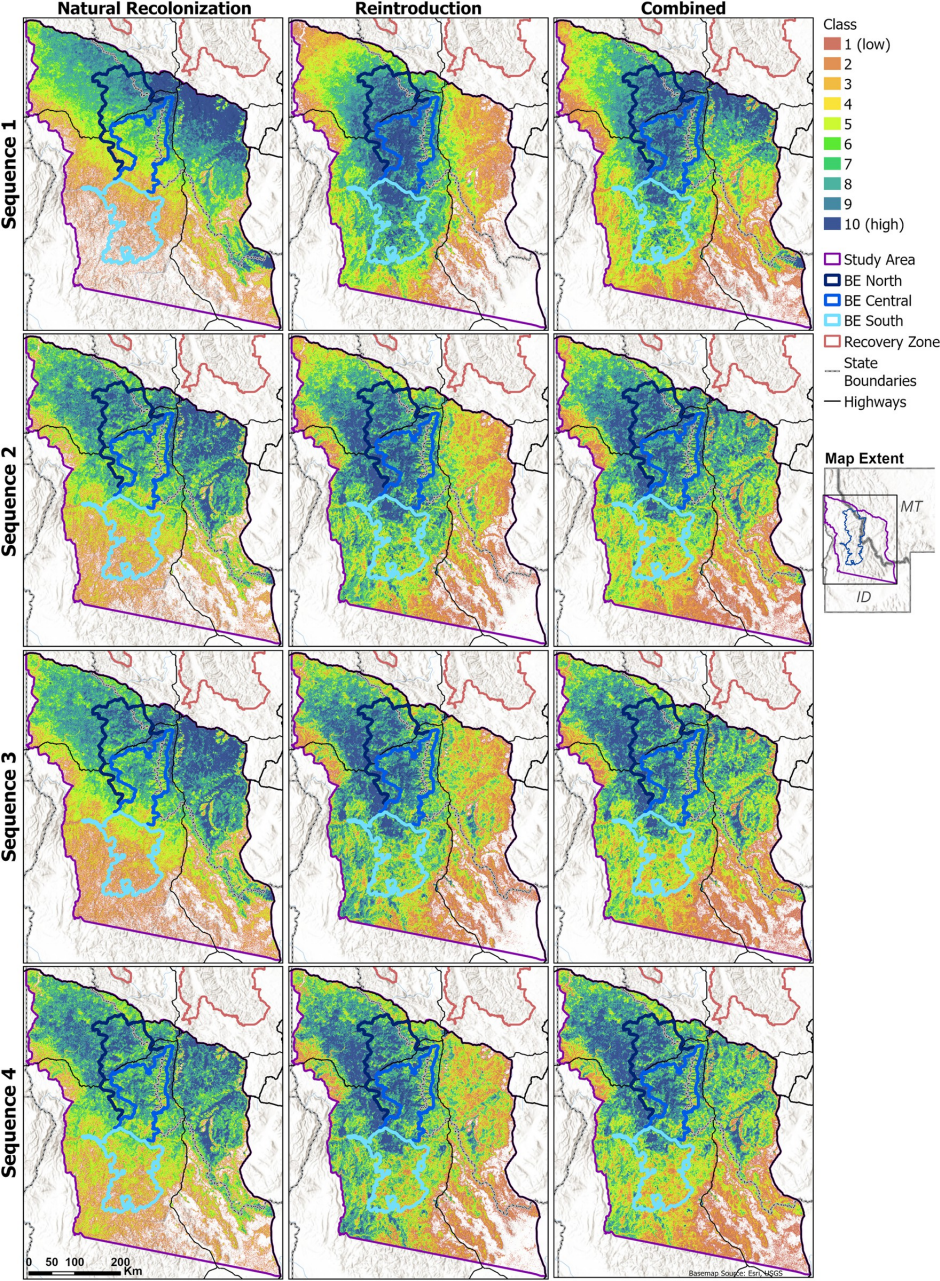
Predicted sequence of habitat use by grizzly bears. Results are shown for the natural recolonization (left panels), reintroduction (middle panels), and combined scenarios. Each map depicts a series of 5,000 steps from the full set of 20,000 steps taken by our simulated bears in each simulation iteration (i.e., Sequence 1 represents steps 1–5,000, Sequence 2 represents steps 5,001–10,000, etc.).

Steps taken by simulated bears during our natural recolonization simulations were widespread within and near the study area, including south of the NCDE Recovery Zone boundary (Fig 3; sex-specific maps are shown in Fig A6 in S1 Appendix). Recolonizing bears were predicted to select most strongly for the northeastern study area; they also more heavily selected for areas west of the GYE and broadly across the northern extent of our study area, including BE North and Central (Fig 4; sex-specific maps and classes binned just for the BE are also shown in Figs A7 –A8 in S1 Appendix). Predicted habitat use for recolonizing bears was concentrated in Sequence 1 (steps 1–5,000) near the start zone and areas closer to existing populations (Fig 5). High predicted use shifted southwestward in Sequence 2 (steps 5,001–10,000), and this continued across Sequences 3 and 4 (steps 10,001–15,000 and 15,001–20,000, respectively). In total within the BE, use was predicted to increasingly concentrate in and near BE North and Central.

### Reintroduction simulations

Steps taken by simulated bears for a reintroduced population occurred throughout the BE and over much of the study area (Fig 3). Highest relative predicted use was concentrated and contiguous in the northwestern two thirds of the study area, including BE North and Central (Fig 4). Predicted habitat use for reintroduced bears was relatively widespread throughout the BE in Sequence 1 (Fig 5). Sequences 2–4 showed progressive shifts towards BE North and Central, and into the areas west of the BE boundary.

Assuming natural recolonization occurred concurrently with a reintroduction into the BE, simulated bears used habitat over most of the northern study area (Figs 4 and 5). This contrasted with use concentrated in the northwestern or northeastern study area under reintroduction or natural recolonization alone, respectively.

### Predictive capacity

Locations of 63 grizzly bear outliers aligned well with the recolonization maps (Table A2, Fig A9 in S1 Appendix). Overlaying the outlier locations onto the map produced a Spearman rank correlation of 0.94 with a mean class value of 9.1. Of the 63 outliers, 52.4% were in the top class (10) and 96.8% were in the top 5 classes (Fig A10 in S1 Appendix).

Locations of GPS-collared bears likewise aligned well with the recolonization maps (Fig A9 in S1 Appendix). In total, GPS locations of 2 females with 463 total fixes and 4 males with 6,348 fixes were available (Table A2 in S1 Appendix). Overlaying the collar locations onto the maps produced a Spearman rank correlation of 0.93, with a mean class value of 9.6. Of the 6,809 total GPS-collared locations, 79% were in the top class and 99.8% were in the top 5 classes (Fig A11 in S1 Appendix).

### Habitat assessments

**Fig 6:**
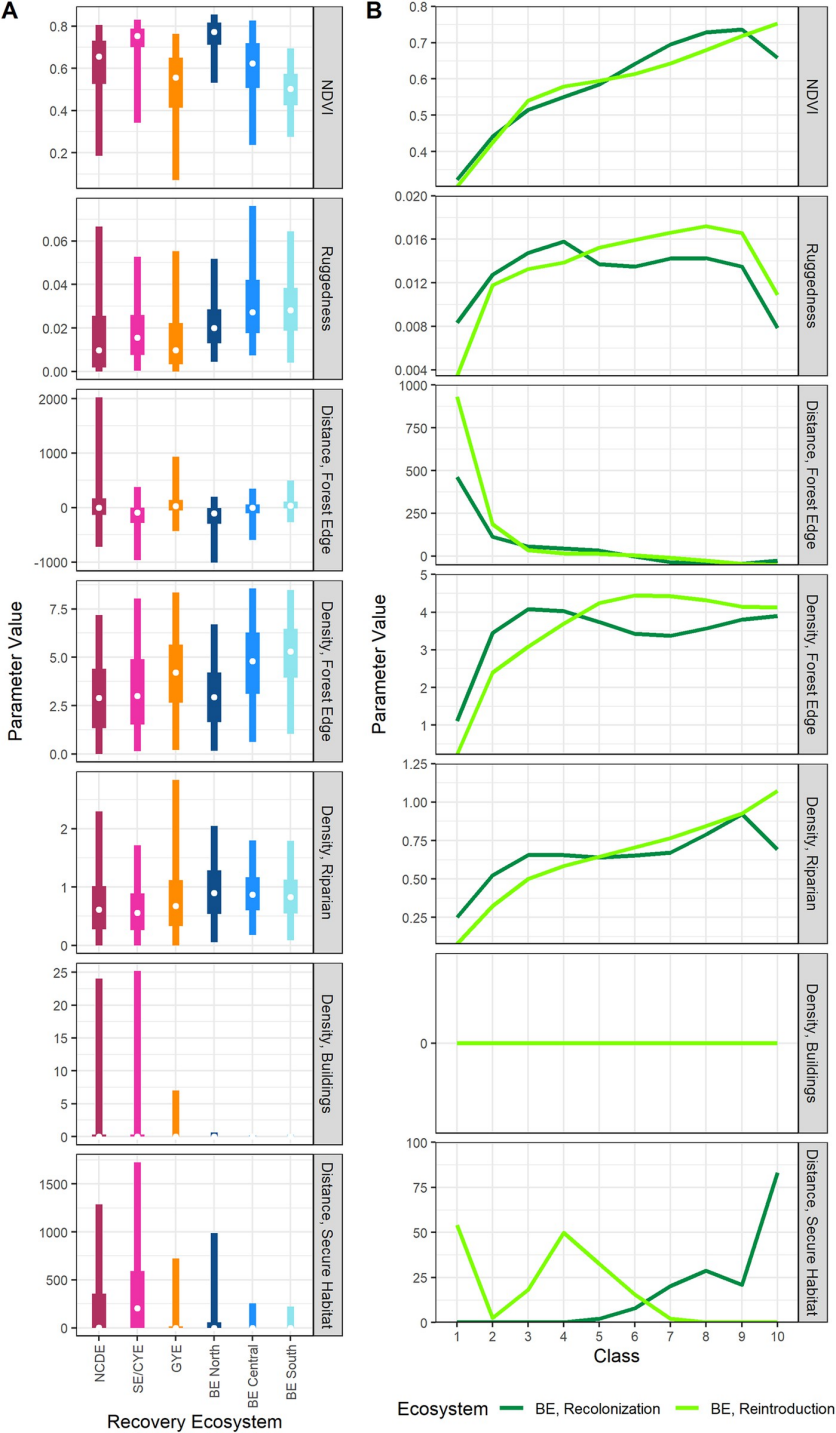
Habitat assessment results. Panel A: comparison of habitat values within the BE and other recovery ecosystems. White dots are median values, boxes are 50% interquartile ranges, and thin lines extend to the 95% values. Panel B: Mean values of habitat variables used by grizzly bears during simulations, measured within each class of predicted habitat use (1 = lowest, 10 = highest) within the BE study area. Where only one line is visible in Panel B’s 6^th^ graph for building density, the recolonization results were identical to the reintroduction results (and thus behind that line); building densities were at or near 0 in the BE.

Habitat available in the BE was broadly similar to habitat in the NCDE, GYE, and SE/CYE (Fig 6). NDVI values in BE North, Central, and South were each respectively comparable to SE/CYE, NCDE, and GYE values. Ruggedness was generally greater in the BE, particularly in BE Central and South. Distance to forest edge was generally lower in the BE yet comparable across ecosystems. Density of forest edge was somewhat higher in BE Central and South than BE North; values were comparable for the NCDE, SE/CYE, and BE North, and for the GYE and BE Central and South. Density of riparian was comparable to other ecosystems but slightly higher on average in the BE, whereas density of buildings and distance to secure habitat were lower than in other ecosystems.

Habitat used by simulated bears were similar under the two simulation scenarios and comparable to habitats used by bears in the NCDE, GYE, and SE/CYE (Fig 6 and Fig A12 in S1 Appendix). Simulated BE bears on average selected for greater NDVI, lower ruggedness, closer distances to and density of forest edge, greater density of riparian, lower buildings densities, and shorter distances to secure habitat.

**Fig 7:**
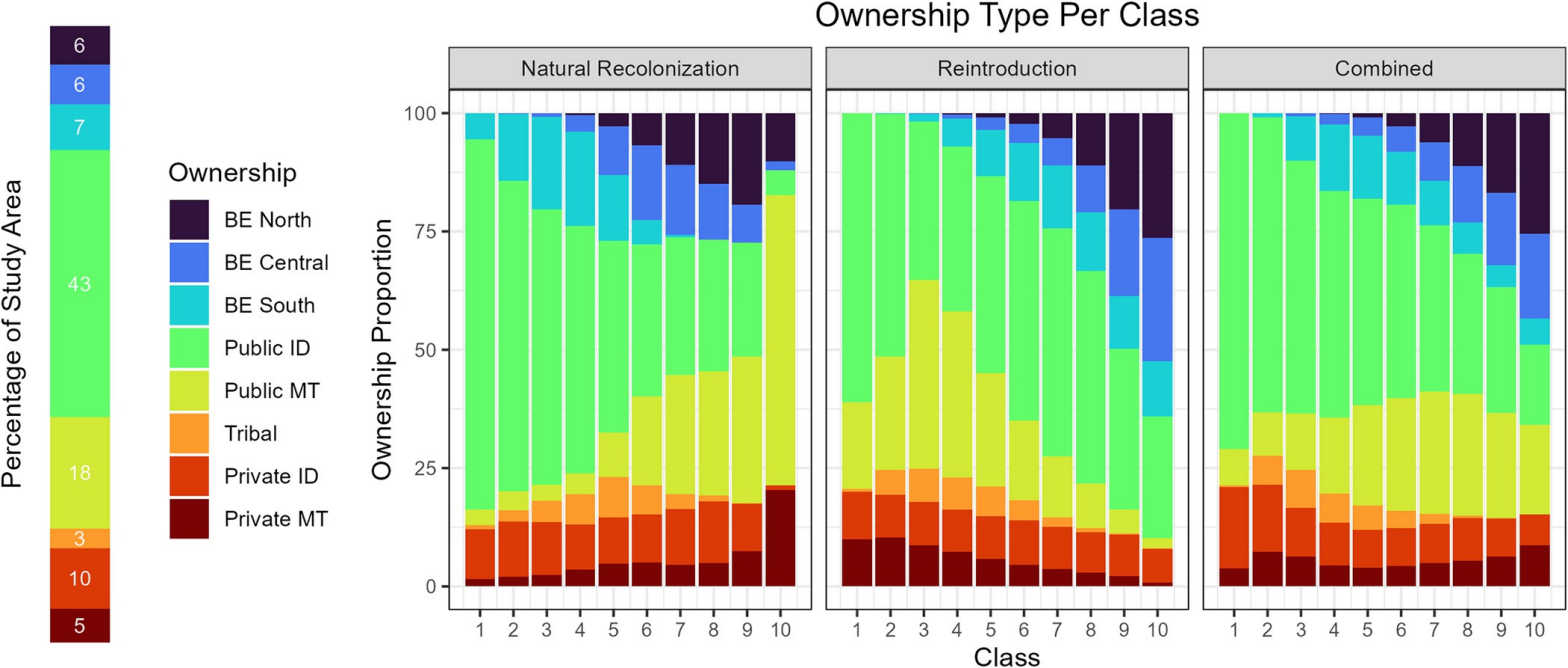
Land ownerships used by grizzly bears. The proportion of ownership or jurisdiction type in the study area is shown on the left side. On the right, the contribution of each type to classes of habitat use (1 = lowest, 10 = highest) by simulated grizzly bears, under recolonization (left), reintroduction (middle), and combined (right) scenarios.

Under the two simulation scenarios, simulated bears used the landscape unevenly by ownership and jurisdiction (Fig 7). Public lands outside of the BE in Idaho (43%) and Montana (18%) made up most of the study area (Fig 1). For the top classes of habitat use, recolonizing bears used public lands in Montana most extensively (Fig 7), followed by private lands in Montana and public lands in BE North, Idaho, and BE Central. Use by simulated reintroduced bears was concentrated in all 3 parts of the BE and nearby public lands in Idaho. Under a combined reintroduction and natural recolonization scenario, bears used the mix of lands across the BE, public lands in Idaho and Montana, and private lands in both states.

## Discussion

Our research aimed to facilitate on-the-ground conservation for grizzly bears in and near the Bitterroot Ecosystem of southwest Montana and central Idaho. Predicted habitat maps are available online at ScienceBase.gov (https://doi.org/10.5066/P91EWUO8). All simulations for grizzly bears under both natural recolonization and reintroduction scenarios indicated that habitat selection was higher in the northern half of the study area compared to the southern half, and higher in BE North and BE Central than in BE South (Figs 4 and 5). This finding matches previous studies, which suggested that drier conditions in the southern areas may reduce productivity or selection of those habitats by grizzly bears [10, 28, 30]. Other than this common north-south gradient, areas of highest predicted habitat selection differed between the two simulated scenarios, even when comparing Sequence 4, representing the most future time frame in our simulations. Under the natural recolonization scenario, highly selected areas were concentrated in Montana during early sequences and then became more uniform across the northern study area during later sequences. In contrast, under the reintroduction scenario, highly selected areas were more concentrated within Idaho, even during the later sequences. Under the assumption of natural recolonization occurring amidst a reintroduction into the BE, use was predicted to remain more widespread across both Idaho and Montana within the northern half of the study area.

Based on simulations of natural recolonization, we expect that as grizzly bears return to their former habitats in the region, relative habitat use will be higher in the north end of the study area and BE (BE North and Central, Fig 1), especially in areas south and southwest of the NCDE on the Lolo, Beaverhead-Deerlodge, and Bitterroot National Forests (Figs 4 and 5). This is expected given the proximity of this area to existing populations in the CYE and NCDE, similarity of habitats, and greater likelihood of starting movements from this area. Although limited data were available for assessing the predictive power of these maps, existing data indicated strong performance, with Spearman rank correlations of ≥0.93 and mean class values at outlier and GPS collar locations of ≥9.1 (Table A2 in S1 Appendix). Furthermore, 52.4% of outlier locations and 79% of GPS collar locations were in class 10 in our predicted habitat maps for natural recolonization. To date, observed movements and outlier locations have occurred primarily in the study area’s north (and especially in the northeast; Fig A9 in S1 Appendix). When population of origin could be confirmed, all recent bears came from the NCDE. Simulations also predicted that most grizzly bears naturally recolonizing westward from the GYE will use the Centennial Mountains (Caribou-Targhee NF) into the Beaverhead and Tendoy Mountains (Beaverhead-Deerlodge NF; Fig 4, Fig A3 in S1 Appendix), along the Idaho-Montana border. The Beaverhead Mountains run diagonally from southeast to northwest into the high-use areas in the northeast study area. Along the way, they pass the crescent-shaped Big Hole Valley (Fig 1). Our simulated bears used parts of this valley extensively (Fig 4). This is an area with numerous observations of grizzly bears in the past decade, including one male that originated from the NCDE (Fig A9 in S1 Appendix).

Our reintroduction simulations predicted that if reintroduced to the BE, grizzly bears would use habitat throughout the BE, but also surrounding areas (Figs 3 and 4). For reintroduced bears, highest use was predicted to be within the BE and public lands northwest of the BE. Lower habitat use was predicted in the eastern third of the study area, particularly the southeastern corner. These results imply that if reintroduced or once established in the BE, grizzly bears from this area would be likely to concentrate use in the BE vicinity, particularly in and near BE North and Central. However, these simulated bears also tended to use public lands westward of the BE boundary, revealing that once bears reach the BE, they may be more likely to move into areas in and near the Idaho Panhandle and Nez Perce-Clearwater NFs rather than eastward past the relatively densely populated Bitterroot Valley (Fig 1). This in turn may mean that a reintroduced or reestablished population would have greater chance for connectivity with the SE, CYE, and NCDE, but less connectivity with the more distant GYE to the BE’s southeast.

Were a reintroduction to occur, natural recolonization is expected to continue as subadult bears disperse from nearby populations. Combining reintroduction and natural recolonization simulations, we predicted grizzly bears to have extensive use within the northern half of the study area, including the region directly south of the NCDE and within BE North and BE Central, along with public and private lands adjacent to these areas (Figs 4 and 5).

Our sequence maps represent predicted changes in habitat use over time (Fig 5). We note that these maps are not meant to predict specific timeframes to achieve population reestablishment. Although our simulations were based on steps at 3-hour (+/- 45 minutes) intervals, they did not factor in rest periods, whereas real bears spend part of each day resting and their movement behaviors are likely to shift across seasons (with no movement during hibernation, which lasts for varying durations). Simulated bears also did not die, whereas real bears would encounter mortality risks, especially from humans and human infrastructure [16, 40–43]. Furthermore, real dispersing bears would eventually set up home ranges where their movements would become localized to an area [5]. Our models included no homing mechanism to explicitly represent home ranging behavior. Despite these limitations, the sequence maps depict how overall space use by naturally recolonizing or reintroduced grizzly bears might shift over time based on these assumptions. These various assumptions also eliminated the need to make additional assumptions about time to recovery, mortality risk, and home range selection, thereby avoiding added model complexity. Areas characterized by contiguous cells with higher class values might be considered potential locations where real bears might select home ranges (with the mortality caveat above). As real bears settle and successfully reproduce, their offspring would then disperse from these sites in the future, potentially following a sequence of shifting habitat use patterns through time as predicted in our simulations.

The finding that simulated grizzly bears in a reintroduced population were relatively unlikely to spend time in the northeastern study area (Fig 4) contrasts with results for recolonizing bears (who originated in this and nearby areas along the I-90 and I-15 interstate highways). The lower use of the northeastern study area indicates that bears may prefer the BE and its vicinity. Additionally, the relatively populous Bitterroot Valley on the BE’s eastern flank may act as a deterrent to bear movement (Fig 1). It may likewise encourage naturally recolonizing grizzly bears to stay in this northeastern corner of our study area (contributing to our natural recolonization results, Fig 4). Concentration of habitat use in this region likely explains the lower selection for secure habitat compared to reintroduced bears (Fig 6). Our previous Phase 3 research predicted that the Bitterroot Valley strongly influenced pathways for bears dispersing between ecosystems, with minimal crossing points selected by simulated bears traversing the valley into the BE [22]. Helping provide safe passage for dispersing grizzly bears into the BE will likely be key for facilitating recolonization and subsequent reestablishment of grizzly bears in the BE.

Based on previous work demonstrating high accuracy and transferability of our movement models [20–22] and our tests using outlier and GPS collar data in this present study, we expect our predictions for the BE to be accurate and reliable. Habitat values within the BE for variables in our models were also generally comparable to values observed in nearby model training (NCDE) and test populations (SE/CYE and GYE; Fig 6). Eventually, information from grizzly bears that might inhabit areas within the study area or BE could be used to validate and further refine the predictive power of habitat mapping efforts like ours, with BE-specific models.

Differences in approaches make it challenging to compare our results to predictions by Boyce and Waller [30] from 2 decades prior, but our reintroduction maps for the BE provide some similarities in predictions. The previous study used data from bears living in the GYE (1989–1997) and a portion of the NCDE (1988–1994) and combined all data from each area into a single model, whereas Sells et al. [20] retained individual models to capture the variation in habitat use inherent among grizzly bears, albeit from a single population. Boyce and Waller [30] also developed seasonal models, whereas Sells et al. [20] found that the active season (May–Nov) models performed better across seasons for NCDE bears. The Boyce and Waller [30] maps only displayed a section of the BE, and binned results into 10 approximately equal ranges of values (rather than areas), further challenging direct comparisons. Their resulting maps for spring habitat predicted that greatest use would occur near the Selway and Salmon Rivers and their tributaries. Our maps corroborate these predictions, but because our maps represent the primary active season, these predictions hold for May–Nov (Fig 4). In contrast, Boyce and Waller [30]’s summer and fall maps predicted that use would concentrate in small patches in higher elevations. By following more recent advice to bin results into 10 equal-area quantile classes [38], our predictions include more area of higher class values than do the maps by Boyce and Waller [30]. Overall, our habitat selection results corroborated previous studies predicting that habitat productivity, and consequently population density, would likely be lower in the southern BE [10, 30].

Our approach provided a rigorous means to predict habitat use for grizzly bears in the BE and nearby areas. However, several caveats should be considered when using our predicted habitat maps. First, Sells et al. [21] noted that lower accuracy of predicted habitat maps for CYE males during Jun–Aug suggested that seasonal models may be helpful for males in summer. If BE males behaved like CYE males, our predicted maps may be less applicable for males in summer months. Sells et al. [21] postulated that the poorer fit of summer predictions for CYE males was explained by high NDVI values in the CYE, which were much higher than in other ecosystems and most of the BE, except BE North (Fig 6). As a second caveat, habitat within BE South had lower NDVI values than BE Central or North, contributing to generally lower predicted use in BE South. Our maps were accurate for GYE grizzly bears that occurred at more southerly latitudes and lower NDVI values [21]; however, future BE grizzly bears could demonstrate greater affinity to areas of relatively low predicted use, e.g., through learned behavior and flexible habitat use, or if bears originate from the GYE with more similar habitat types.

As noted above, a further caveat is that our simulations do not account for mortality risk, which we would expect to be higher in areas of higher road density and human development [41–43], a factor especially important when considering the natural recolonization simulations. Relatedly, our simulations also assume that models from our source bears in the NCDE suitably capture responses of bears to humans and human infrastructure. We do not expect that all individuals respond to human presence in the same manner. Many of our modeled individuals lived near human developments, while others did not. We therefore expect our models to reasonably capture how a bear population is likely to interact with anthropogenic features. Where our maps show high use predicted to overlap with areas heavily influenced by humans, we acknowledge that actual presence of bears would likely be lower due to the higher mortality risk associated with human presence, including higher incidence of human-bear conflict. Nevertheless, our predictions showing that grizzly bears select habitats in close proximity to human developments, despite the associated mortality risks, are supported by previous studies such as Lamb et al. [16] who described the behavioral and demographic mechanisms underlying the presence of grizzly bears in human-dominated landscapes in British Columbia, Canada. Our results are further supported by substantial documented use of habitats in or near towns and cities. For example, grizzly bears in the NCDE population overlap with a human population of >100,000 people in Flathead County, Montana, including the cities of Kalispell, Whitefish, and Columbia Falls.

Since our models lacked mechanisms to explicitly simulate selection of home ranges by bears, we acknowledge that our results may have overestimated the full extent of bear habitat use in the region. On the other hand, natural recolonization and reintroduction both involve bears using unfamiliar, novel environments initially lacking other resident bears; therefore, the wide-ranging movements of simulated bears may be realistic. Scientists have observed that naturally recolonizing, relocated, and reintroduced bears often range widely [44–47], as do other carnivores. For example, Devineau et al. [48] examined patterns of movement and survival of 218 lynx (*Lynx lynx*) following their reintroduction to southern Colorado during 1999–2006, one of the largest carnivore reintroduction programs to date. Comparing their movements to the large area of contiguous high-elevation habitat surrounding the release sites (20,684 km^2^), they found that 81% of lynx spent time outside of this area and 28% spent more time outside than inside the area. Lynx ranged widely across much of western Colorado and long-range movements by some individuals were observed in 11 surrounding states, with distances up to 1400 km from the release sites.

The process for reestablishing a self-sustaining grizzly bear population in the BE region will be unlike recovery of the populations in the SE, CYE, NCDE, and GYE. Those recovery zones were designated in areas where remnant populations still existed. Research has shown that sizable remote areas and reduced vehicular access of recovery zones have been essential for ensuring low mortality rates and long-term persistence of populations [42, 43]; therefore, it is understandable that similar attributes became the focus of planning for the BE [10]. With its large wilderness areas and extensive multiple-use public lands, the BE conforms to this blueprint. Nevertheless, simulated grizzly bears selected habitats over a much larger landscape than the BE itself, under both natural recolonization and reintroduction scenarios.

We emphasize that a vital component of the planning process for grizzly bear recovery in the BE may be *not to assume* that they will be most attracted to the designated BE. Our results indicate that many of the habitat characteristics grizzly bears seek (e.g., forest edges, riparian areas) for both home ranges [20, 21] and movement pathways [22] were also present in areas surrounding the BE, including on other multiple-use and private lands. In recent decades, as existing grizzly bear populations have expanded, they have increasingly used private lands [49]. Cover and food resources in river valleys and surrounding foothills and mountains have been shown to be attractive to grizzly bears, even where higher mortality risk results in a sink or ecological trap paradigm [16, 40]. Other examples exist where at-risk species show high use and even selection for resources outside of protected areas, such as cheetahs (*Acinonyx jubatus*) [50, 51] and Asian elephants (*Elephas maximus*) [52].

Given that the conservation value of a reestablished BE grizzly bear population [9] stems not only from its existence as another population (i.e., ensuring redundancy), but also from its capacity to function as part of an interconnected metapopulation in the lower 48 states (i.e., promoting resiliency), our results highlight the prospect of at least some presence of grizzly bears in more human-dominated landscapes between recovery areas. Recognizing and planning for the role of private lands in the long-term conservation of grizzly bears will benefit grizzly bears and humans alike, as will taking appropriate steps to make landscapes work for both humans and grizzly bears. This will require significant financial resources and personnel to work directly with private landowners to help prevent human-bear conflict and respond promptly to conflict when it occurs, in effort to help increase the acceptance of grizzly bear presence over a large landscape.

## Supporting information

S1 Appendix Predicting future grizzly bear habitat use in the BE. (DOCX)
