# Supplementary material for: Predicting future grizzly bear habitat use in the Bitterroot Ecosystem under recolonization and reintroduction scenarios
Source: PLoS One. 2024 Sep 4;19(9):e0308043. doi: 10.1371/journal.pone.0308043 (PMC11373846; doi:10.1371/journal.pone.0308043)
Supplement: S1 Appendix — (DOCX) [file pone.0308043.s001.docx]

**SI Appendix. Predicting future grizzly bear habitat use in the Bitterroot Ecosystem under recolonization and reintroduction scenarios**

Sarah N. Sells and Cecily M. Costello

**Additional Details**

Our study builds on Sells et al. [1]. The methods used in this earlier study are detailed below (see also Fig 2, main text) and are available online at <https://doi.org/10.1016/j.biocon.2022.109813>.

***NCDE Description***

Models used in our analysis were derived with data from GPS-collared bears living in the nearby Northern Continental Divide Ecosystem (NCDE) recovery zone and its surrounding Demographic Monitoring Area (demarcating the extent of population monitoring, totaling 42,625 km^2^; Fig 1). The core of the NCDE was characterized by high rugged mountains, where forested areas transition to high alpine zones that can have extensive snow and ice. Areas west of the Continental Divide were strongly influenced by a maritime climate, whereas areas east of the Divide had a continental climate. The western edge of the NCDE was densely forested with rugged mountains. The east edge of the NCDE included the edges and foothills of the Rocky Mountains, which transitioned further east to plains, wetlands, and long, narrow riparian corridors. The southern edge of the NCDE was mountainous with forested areas and alpine zones at higher elevations, foothills covered in grasses, shrubs, or woodlands, and intermontane valleys with grasses or shrubs.

***GPS Data***

Sells et al. [1] used location data from grizzly bears in the NCDE to develop integrated step selection functions (iSSFs) [2, 3]. From 2003 – 2020, grizzly bears were captured using culvert traps or foot-hold snares, collared with Telonics GPS transmitters, and aged from a premolar tooth [4] or based on tooth eruption, wear and coloring.

Sells et al. [1] processed grizzly bear GPS data in Program R [5] using package amt [3]. They included data for May – November (to avoid denning months) and omitted dates when bears were trapped, released, or killed. After filtering fixes to a consistent time interval [2] of 3 hours (+/- 45 minutes), they retained steps of 100 – 15,000 m (omitting stationary and suspect steps), and all bears with ≥100 steps and ≥100 days of steps available. They paired each used step with 10 control steps from the same starting point but with step lengths and turn angles drawn randomly from each individual’s gamma and von Mises distributions, respectively, as summarized from their movement data [3].

For each bear step (used and control), Sells et al. [1] measured the Normalized Difference Vegetation Index (NDVI), terrain ruggedness, distance to forest, density of forest edge, density of riparian, density of buildings, and distance to secure habitat (Table A1). They prepared datasets as rasters with 300 m resolutions. To index food availability, they used package MODIStsp [6] to obtain data during peak green-up (Jun 15 – Jul 15) [7] each year from 2005 – 2020, which was then averaged at each raster cell. They used package elevatr [8] to access elevation data, and spatialEco [9] to calculate ruggedness from elevation data as the vector ruggedness measure [10]. For distance to forest edge, Sells et al. [1] reclassified the 2016 National Land Cover Dataset (mrlc.gov) to forest (deciduous, evergreen, mixed forests, and woody wetlands) and non-forest (remaining classes), identified forest boundaries with package raster [11], and measured Euclidean distance. For density of forest edge, they measured forest edge per km^2^. For density of riparian, they measured line density per km^2^ for waterbody boundaries, rivers, streams, and artificial paths outside waterbody boundaries in the National Hydrography Dataset (U.S. Geological Survey, 2020, National Hydrography Dataset (ver. NHD 20200615), accessed June 20, 2020 at www.usgs.gov/national-hydrography/access-national-hydrography-products). They measured ruggedness, density of forest edge, and density of riparian using moving windows with radii of 1100 m (females) and 1500 m (males) from the cell centroid to represent typical daily movements [12]. For density of buildings, Sells et al. [1] calculated centroids of each building footprint in the Microsoft Buildings Footprint dataset (github.com/microsoft/USBuildingFootprints), and measured point density per km^2^. For distance to secure habitat, they measured Euclidean distance to polygons identified as grizzly bear secure core (areas >500 m from roads on federal, state, and tribal lands) [13].

## iSSFs

Sells et al. [1] used iSSFs to develop habitat models for grizzly bears in the NCDE to mechanistically predict space use [2]. iSSFs compare covariates associated with animal locations and randomly selected nearby locations using a likelihood equivalent of a Cox proportional hazards model to estimate conditional selection coefficients. The iSSF has exponential form of *w*(x) = exp(xβ), where *w*(x) is the iSSF score, x is a vector of habitat covariates, and β is the coefficient vector estimated via conditional logistic regression. Higher iSSF scores indicate greater relative probabilities of selection.

Sells et al. [1] used Program R [5] and package amt [3] to prepare a predictive iSSF for each bear for use in simulating habitat selection. They internally evaluated predictive capacity of a global candidate model for each bear (all habitat covariates as quadratic terms) using 100 iterations of 25% testing data and 75% training data via k-fold cross-validation [14]. They then gradually eliminated or re-added terms to determine which model formulation (global or reduced) maximized cross-validation scores for each bear.

After identifying the top model for each individual bear, Sells et al. [1] used the models as detailed in the main text to run simulations.

**Table A1.** Hypotheses, predictions, and variables for grizzly bear habitat selection, tested by Sells et al. [1]. These variables constitute the predictive movement models for the present study.

| Variable | Predicted relationship | Alternative prediction | Sells et al. [1] result |
| --- | --- | --- | --- |
| Normalized difference vegetation index (NDVI) | Positive (to increase access to foods) | Uncertain or negative relationship (if access to high local food availability) | Extensive variability among individuals (i.e., support for both predictions). Females particularly select for NDVI. |
| Terrain ruggedness | Negative to intermediate (to balance energy expenditures versus security from humans and dominant bears) | Positive (to obtain seasonal resources or maximize security) | Extensive variability among individuals. Males were more likely to avoid areas of higher ruggedness. |
| Distance to forest edge | Negative to intermediate (to balance security versus access to food) | Strongly negative (for security from humans or dominant bears) | Extensive variability among individuals. Females were more likely to avoid large distances from forest. |
| Density of forest edge | Positive (to increase access to foods) | Negative (for security from humans or dominant bears) | Extensive variability among individuals. Females in particular used areas of greater densities of forest edges. |
| Density of riparian | Positive (to increase access to food, thermal cover, and water) | Uncertain or negative relationship (to avoid dominant bears or if access to high local riparian densities) | Extensive variability among individuals. Males particularly selected for riparian. |
| Density of buildings | Negative (to decrease exposure to human risk) | Positive (to access anthropogenic food resources) | Variability among individuals. Both sexes generally avoided buildings. |
| Distance to secure (unroaded) habitat | Negative (to increase security from human risk) | Uncertain or positive effect (if access to high local levels of secure habitat, or if conditioned to human presence) | Variability among individuals. Some bears avoided areas further from secure habitat. |

Citations: [15-17]

**Table A2.** Outlier and GPS collar data for grizzly bears in the study area. Mean class values were based on sex-specific maps for GPS data.

| Data type | Source Population | Capture details | Sex | N locations | Timing | Duration | Mean Class |
| --- | --- | --- | --- | --- | --- | --- | --- |
| Outlier | Unknown^1^ | NA | Unknown | 63 | 2010 – 2023 | Unknown | 9.1 |
| GPS | NCDE | Conflict response | Female | 358 | Spring 2014 | 58 days | 8.2 |
| GPS | NCDE | Captured in study area | Female | 105 | October 2022 | 6 days | 8.7 |
| GPS | NCDE | Conflict response | Male | 4,183 | May – Aug 2021 | 87 days | 9.6 |
| GPS | NCDE | Captured in study area^2^ | Male | 274 | Oct 2022 – Oct 2023 | 9 days, 32 days, 3 days | 8.5 |
| GPS | NCDE | Captured in study area | Male | 642 | Aug – Dec 2023 | 122 days | 9.2 |
| GPS | NCDE/CYE | Augmentation to CYE^3^ | Male | 1,249 | May – Oct 2019 | 145 days | 8.1 |

1. A small subset of outlier bears have been DNA genotyped and found to have come from the NCDE.
2. This bear left the study area in October 2022, returned in August 2023 for 32 days, and again in October 2023 for 3 days.
3. This bear was moved from the NCDE to the CYE in 2018 for population augmentation and subsequently moved into the study area in 2019, where he spent 145 days before returning north.


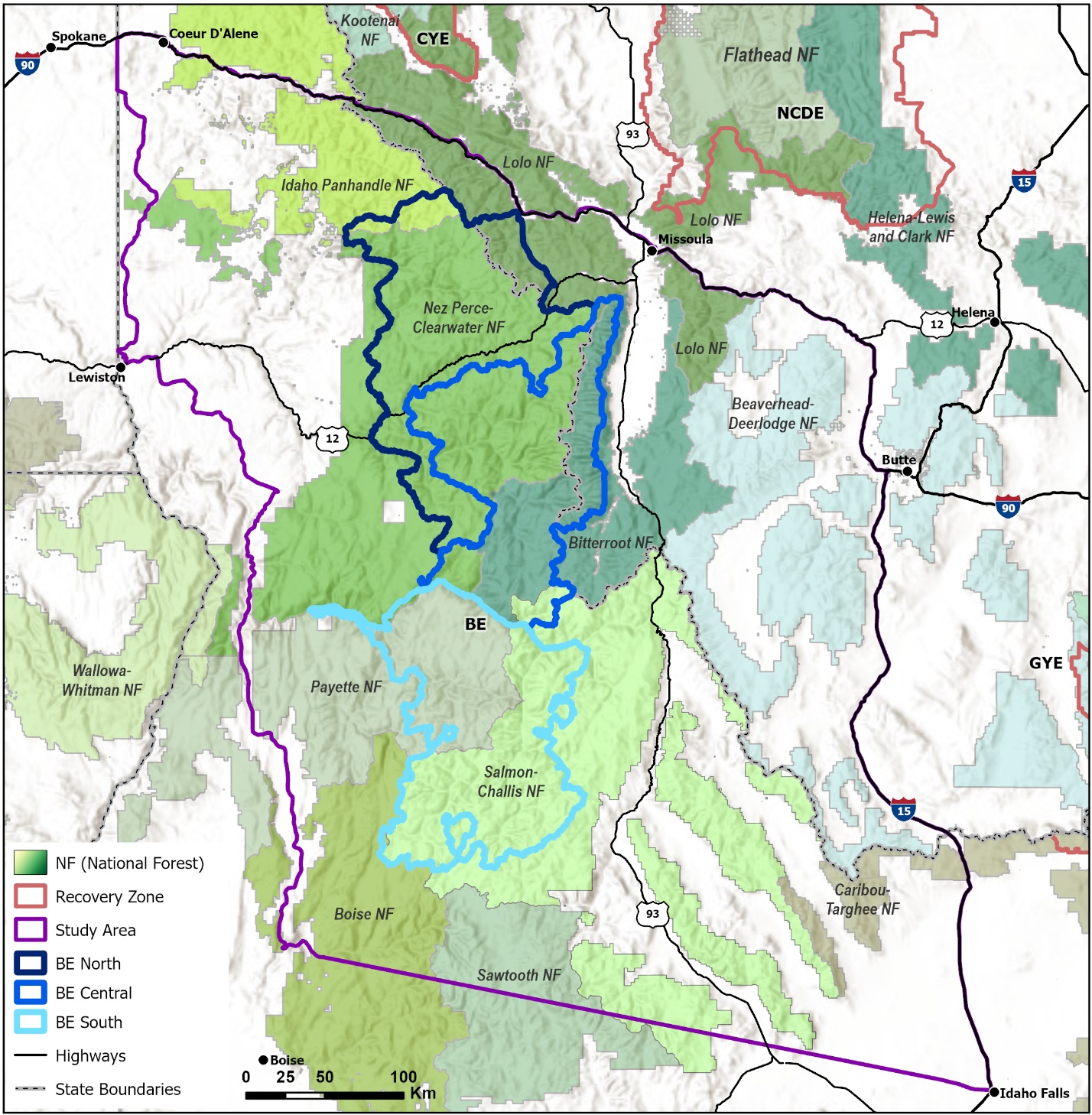
**Figure A1.** National forests (NF) within the study area.


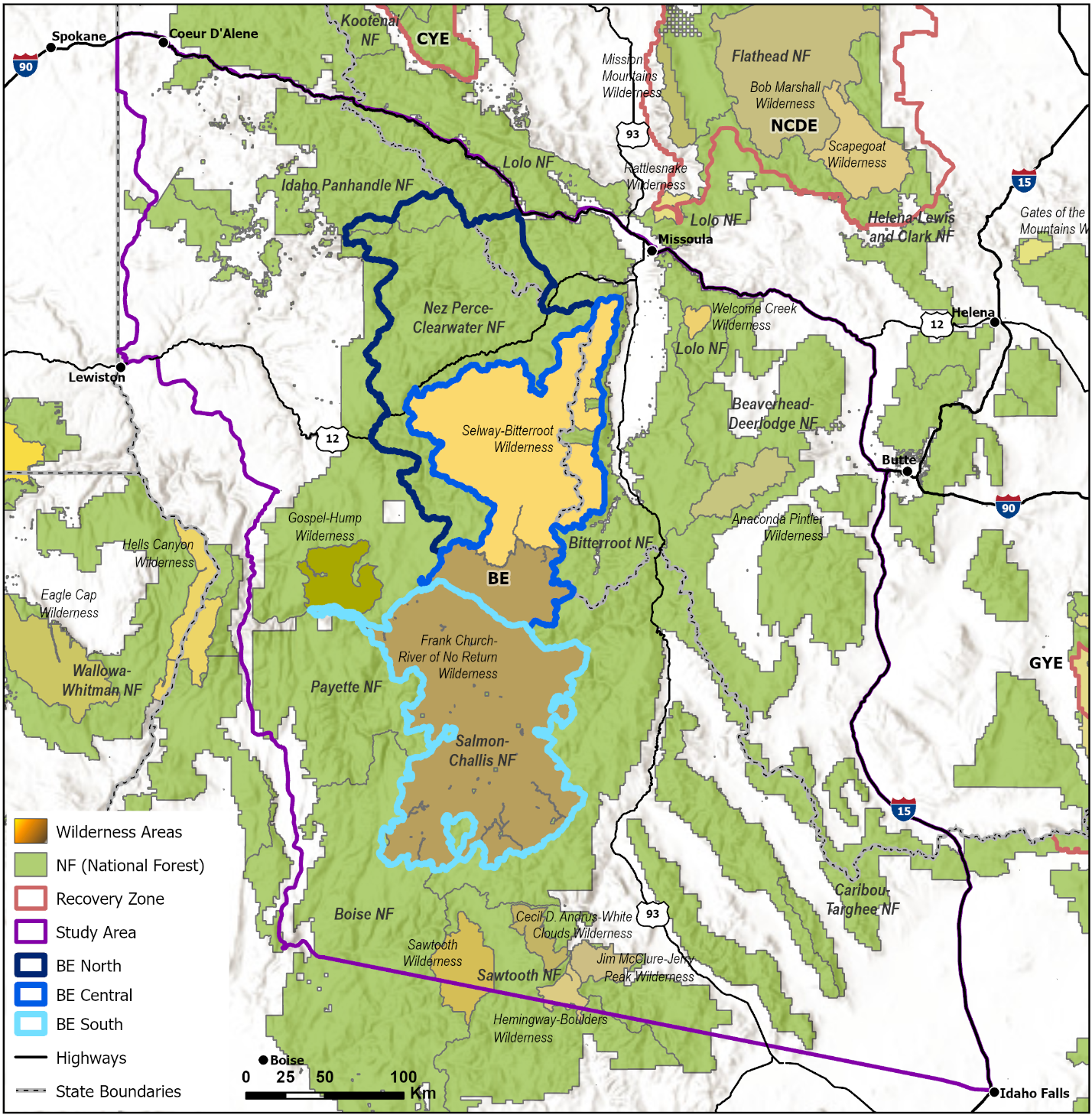
**A2.** Wilderness areas in the study area.


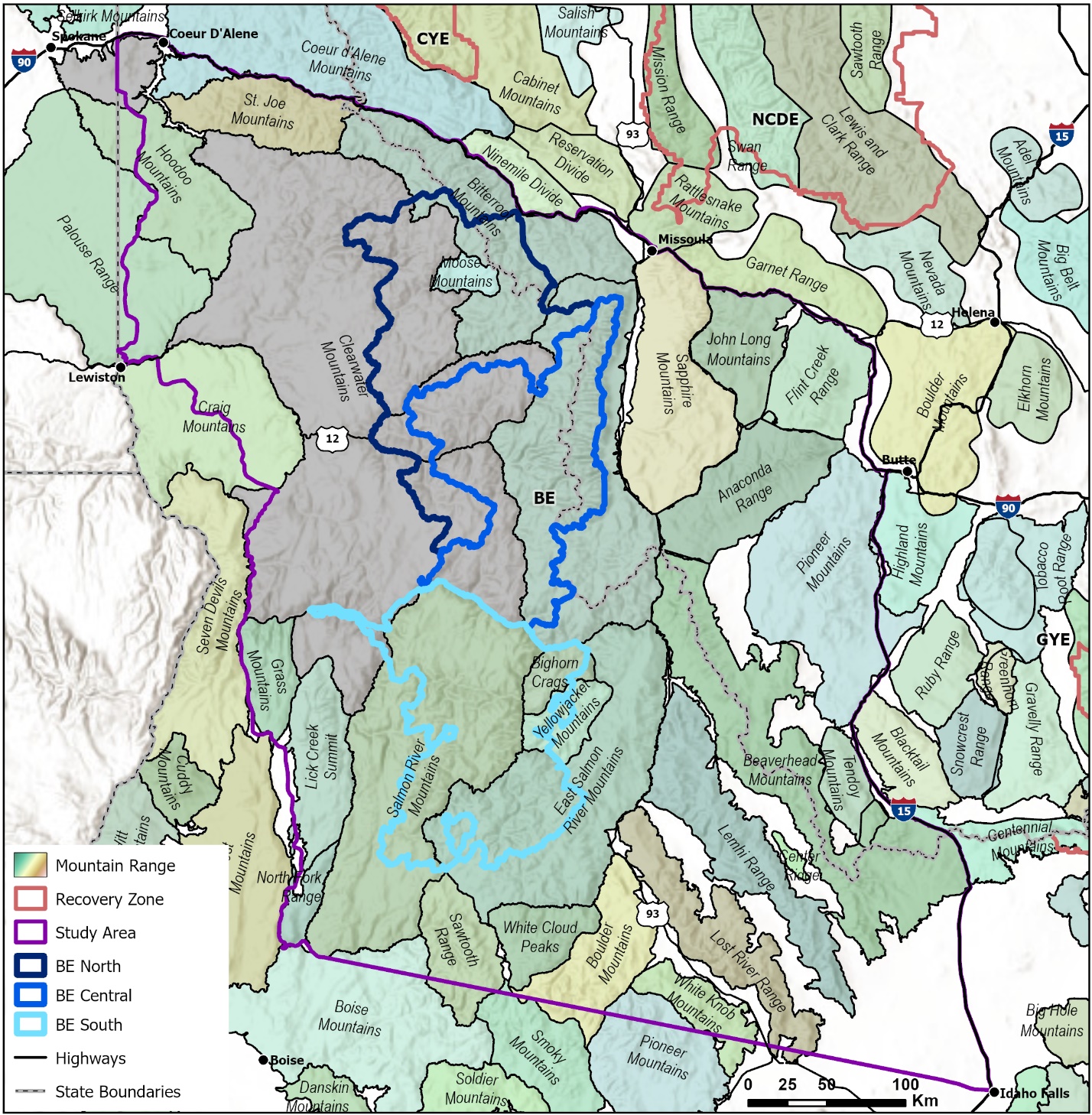
**A3.** Mountain ranges in the study area.


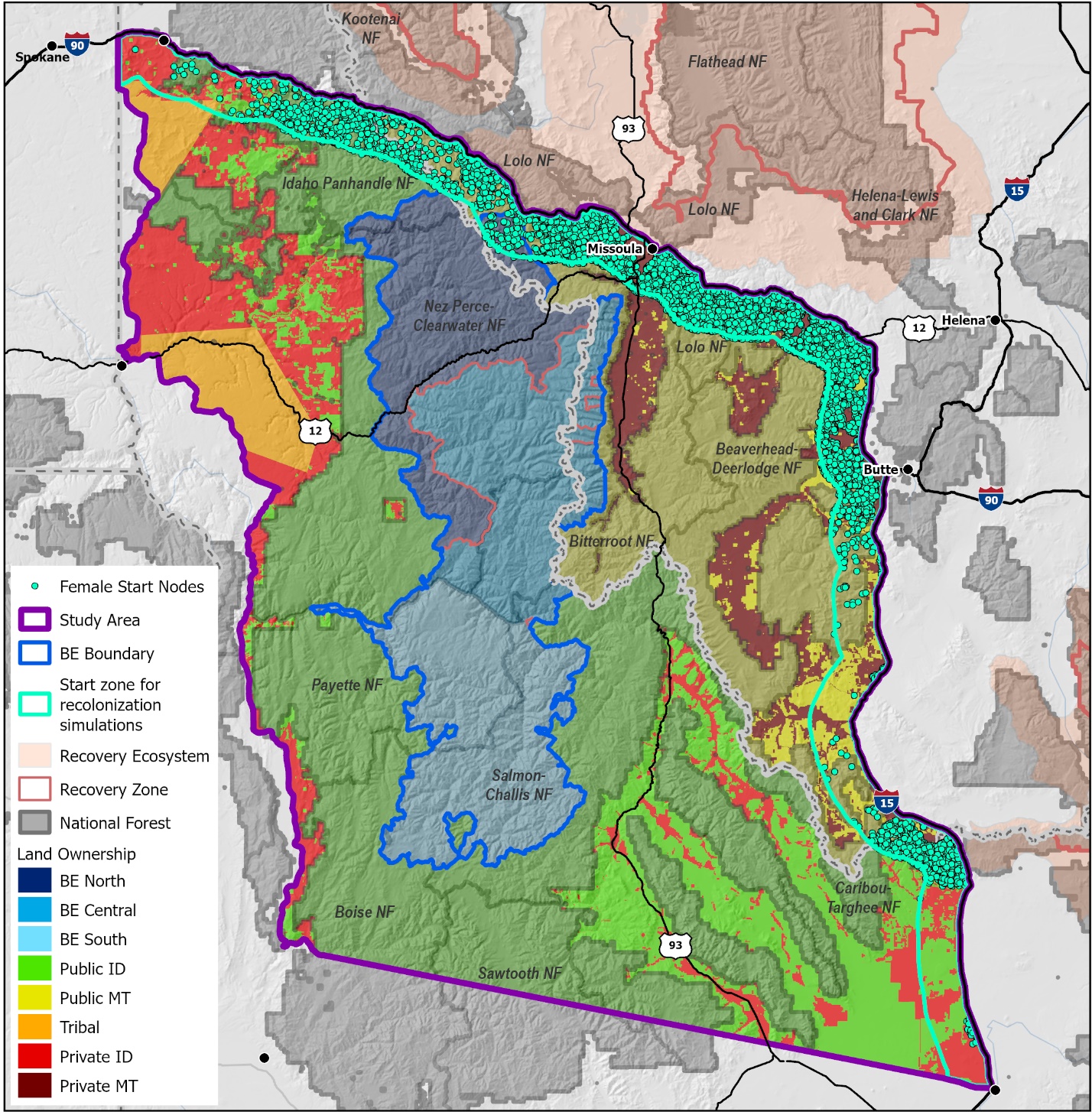
**A4.** Start nodes for females during recolonization simulations. Start nodes occurred in cells with high relative predicted female use (classes 8 – 10) based on undirected simulations in Phase 3 [18].


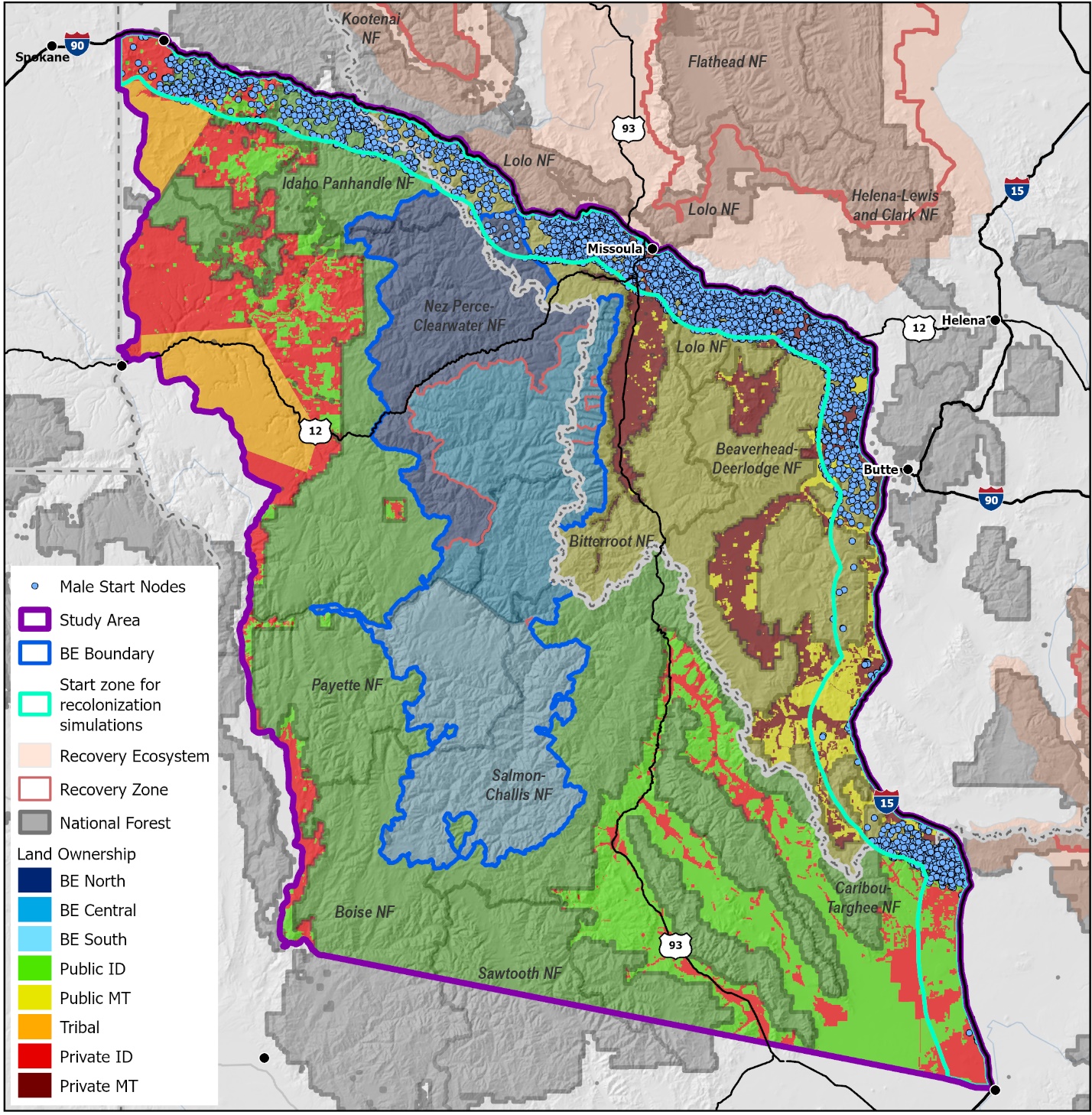


**A5.** Start nodes for males during recolonization simulations. Start nodes occurred in cells with high relative predicted male use (classes 8 – 10) based on undirected simulations in Phase 3 [18].


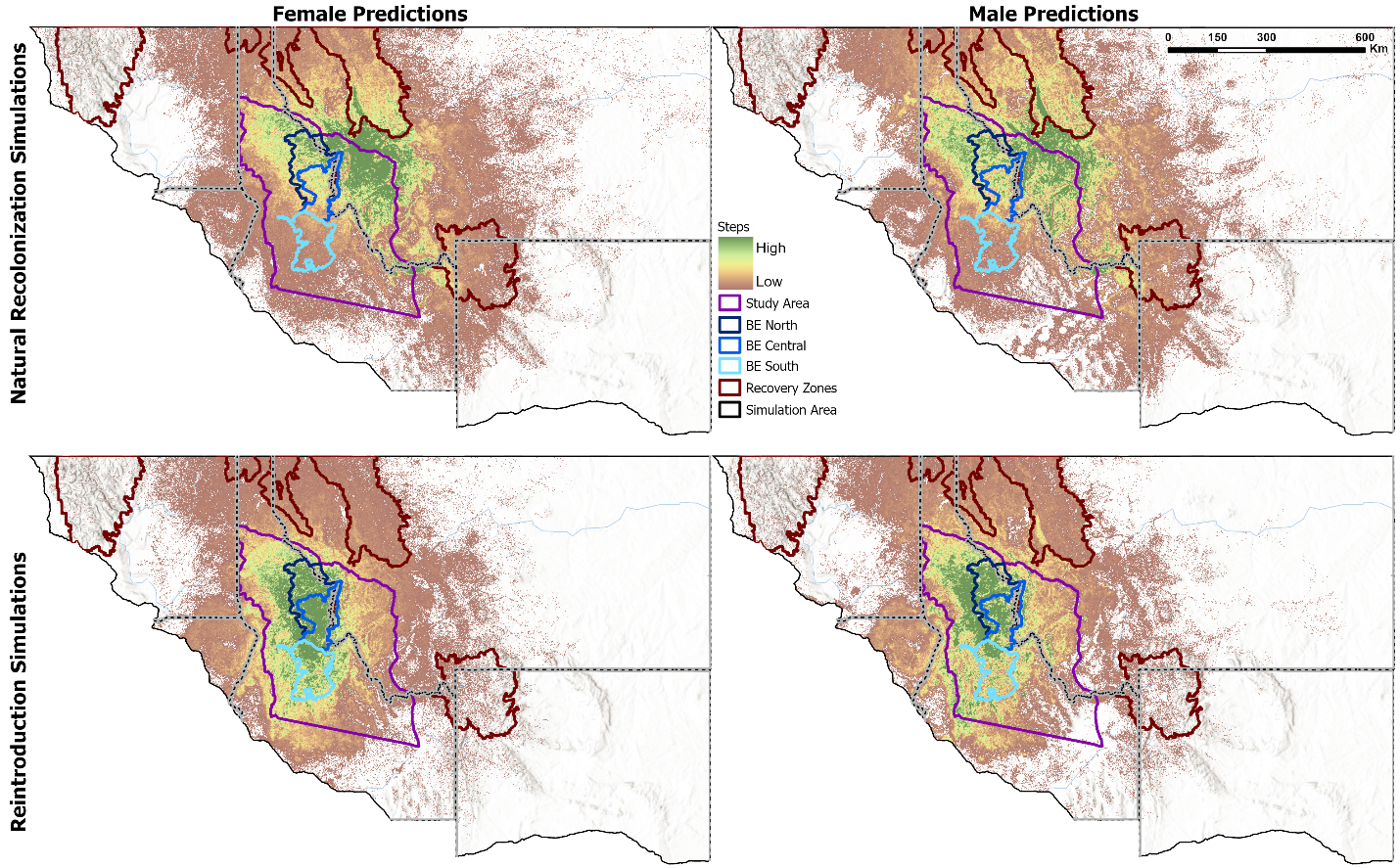


**A6.** Maps of steps taken during simulations based on models for females (left panel) and males (right panel). Simulations were initiated on the north and eastern edges of the study area for the natural recolonization scenarios (top row) and within the BE for the reintroduction scenarios (bottom row).


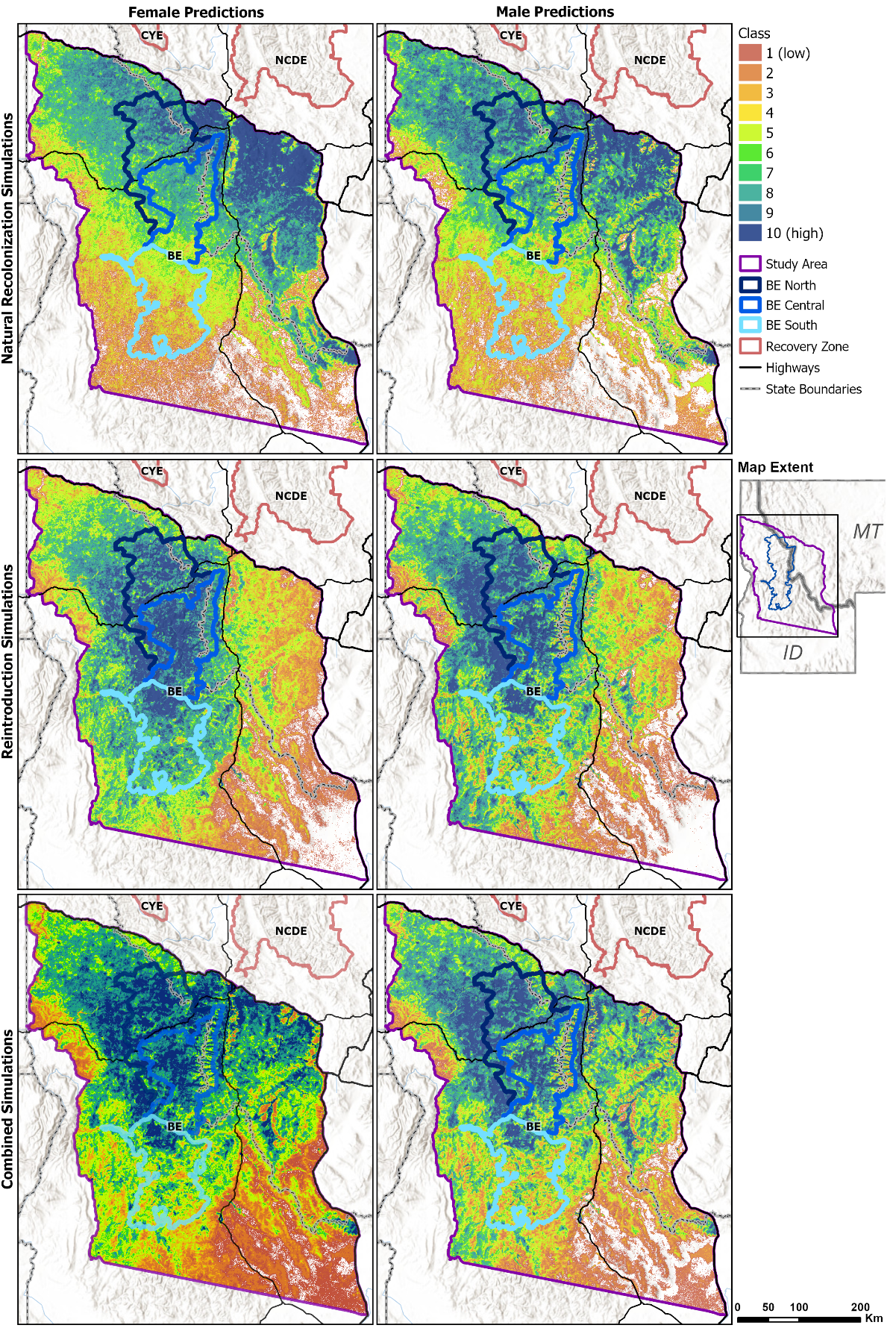


**A7.** Maps of predicted habitat use for female (left panel) and male (right panel) grizzly bears in our study area for the natural recolonization (top row), reintroduction (middle row), and combined (bottom row) scenarios. Classes represent the quantile-binned relative habitat use values (1 = low, 10 = high), as summarized within the study area based on the number of steps taken per 300 x 300 m grid cell (Fig A6).


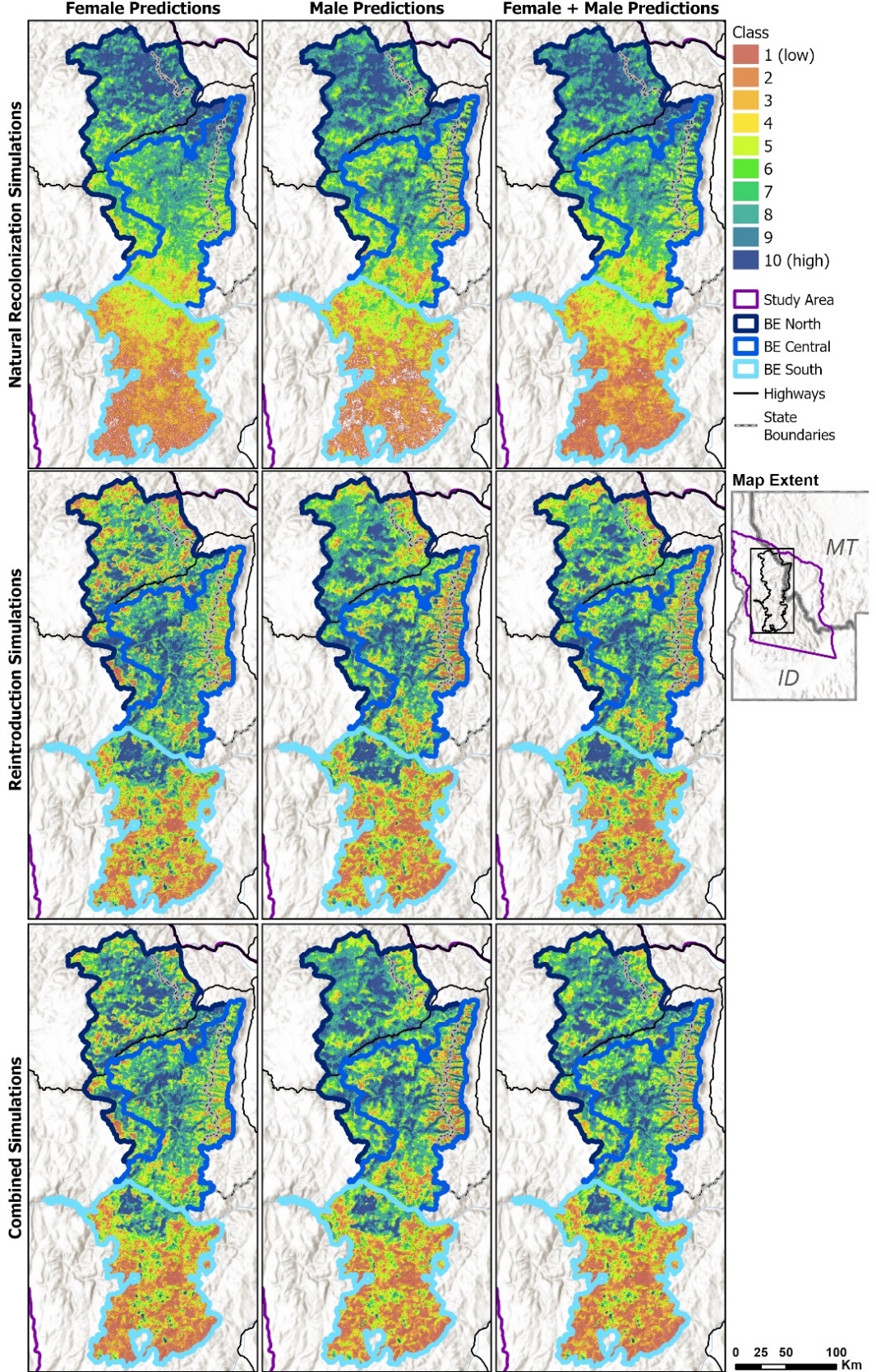


**A8.** Maps of predicted habitat use for female (left panel) and male (middle panel) grizzly bears in the BE for the natural recolonization (top row), reintroduction (middle row), and combined (bottom row) scenarios. “Female + Male Combined” predictions are the merged results for males and females (as also in the main text and results). Combined Simulations represent the merged results for the natural recolonization and reintroduction simulations. Classes represent the quantile-binned relative habitat use values (1 = low, 10 = high), as summarized within the BE (rather than the entire study area), based on the number of steps taken per 300 x 300 m grid cell (Fig 3, main text).


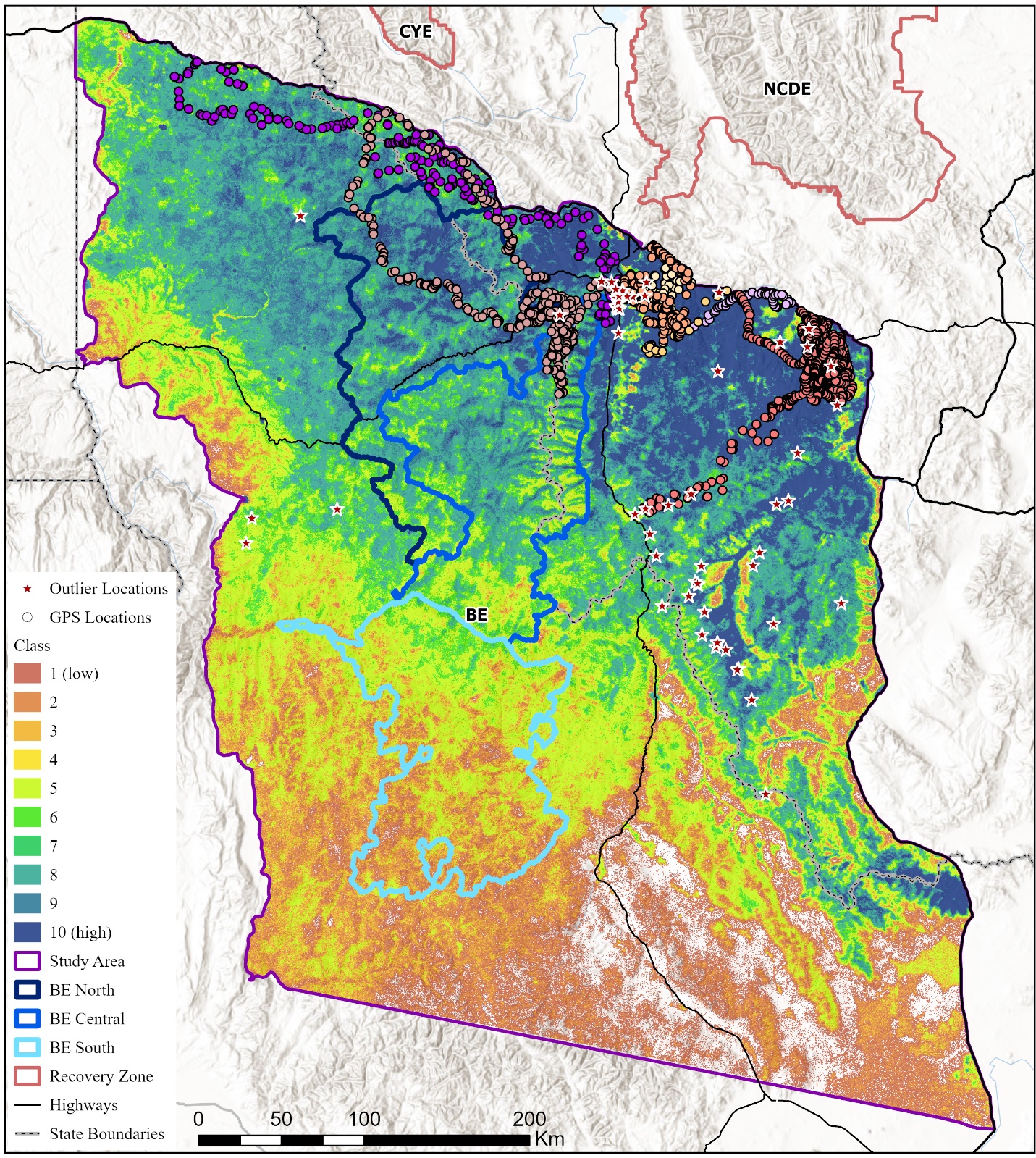


**A9.** Locations of grizzly bear outlier locations and GPS-collared bears within the study area, 2010–2023 compared to simulations results under the natural recolonization scenario.

**
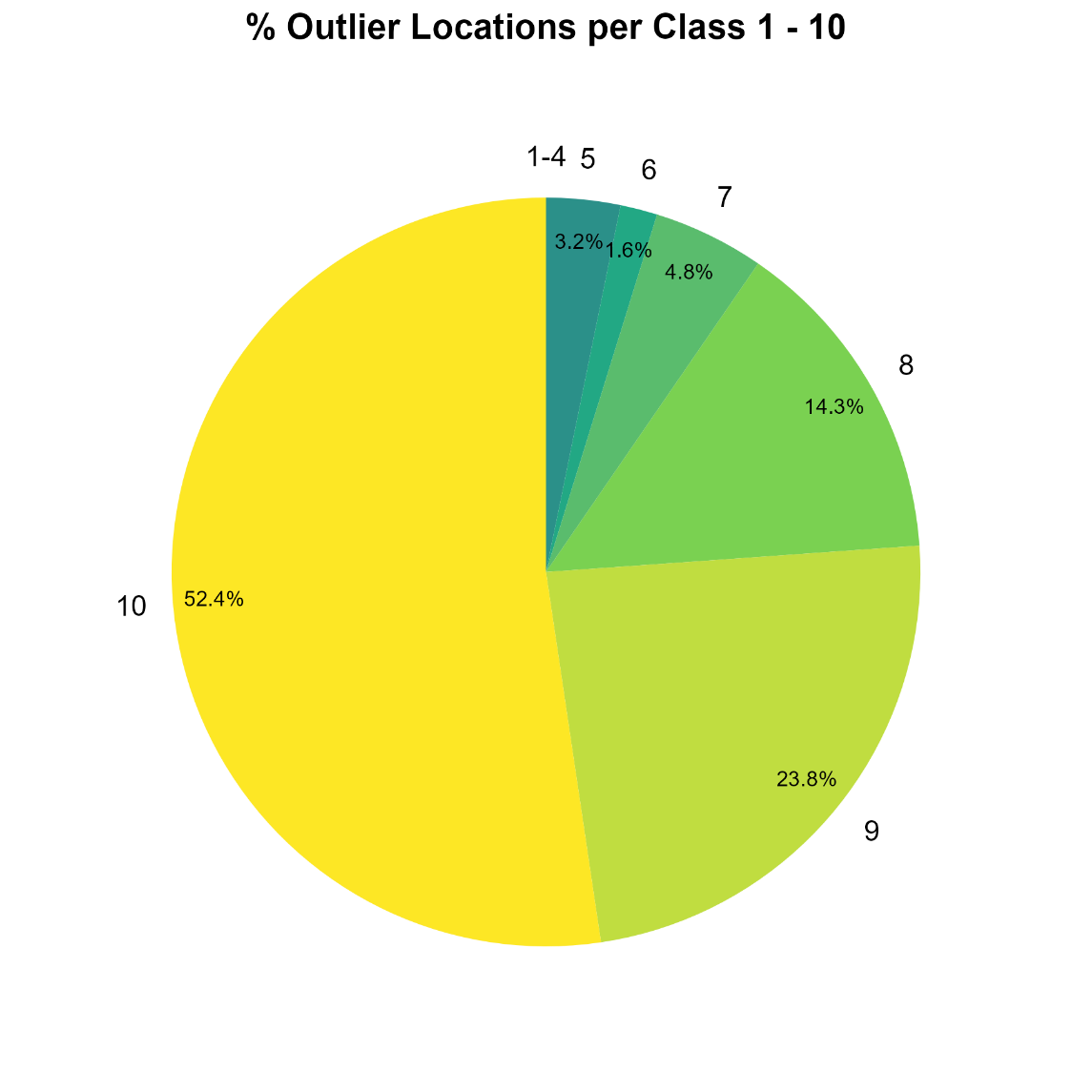
**

**A10.** Percentiles of outlier locations falling within each class of relative predicted use (1 = lowest, 10 = highest) for the study area recolonization maps. (Classes 1 – 4 had no outlier locations.) Spearman rank correlations were 0.94. The mean class value at outlier locations was 9.1, indicating strong performance of the maps in relation to outlier locations.

**
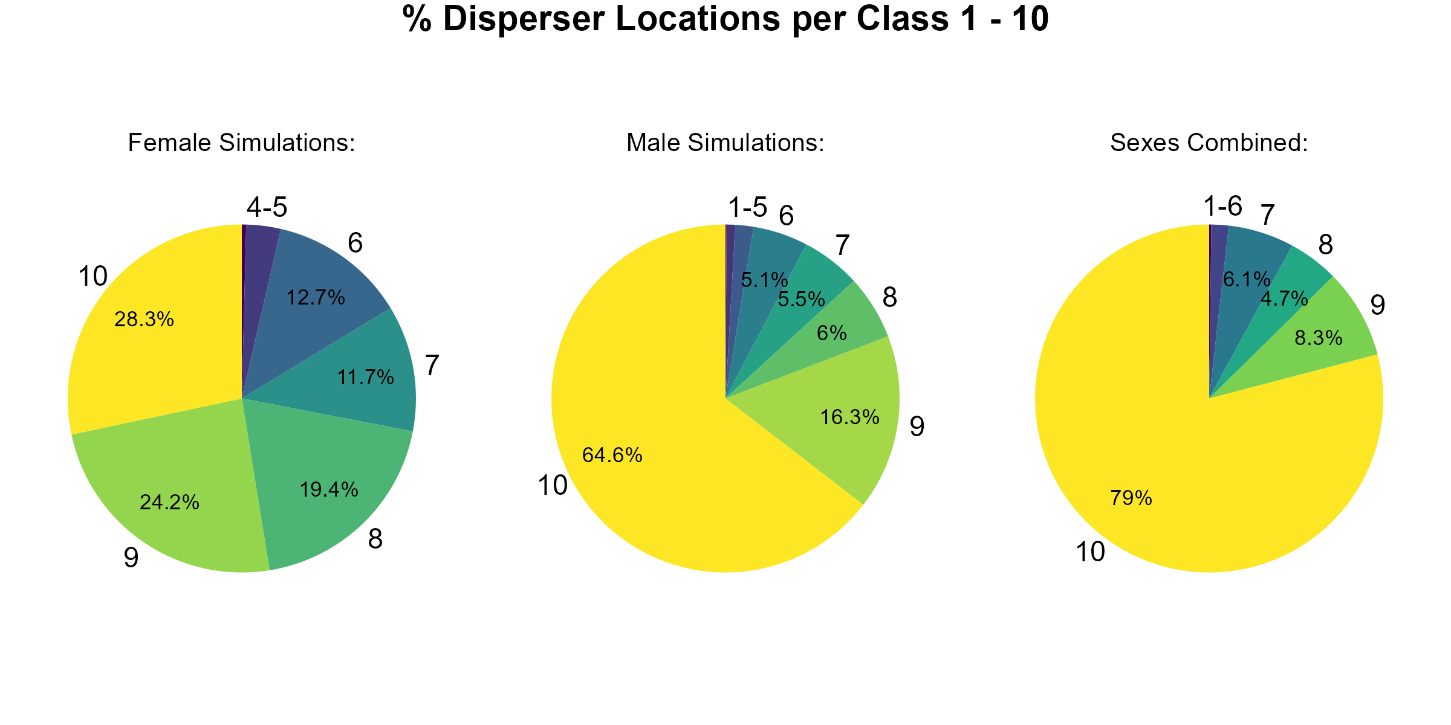
**

**A11.** Percentiles of GPS-collared bear locations (n = 6 individuals, Table A2) falling within each class of relative predicted use (1 = lowest, 10 = highest) for the female and male study area recolonization maps, and for the combined map for both sexes. Spearman rank correlations were 0.97 for the female fixes (n = 463) on female maps, 0.99 for male fixes (n = 6,345) on male maps, and 0.93 for the map of sexes combined. The mean class value at outlier locations was 8.3 for female maps, 9.2 for male maps, and 9.6 for the combined map, indicating strong performance of the maps in relation to outlier locations.


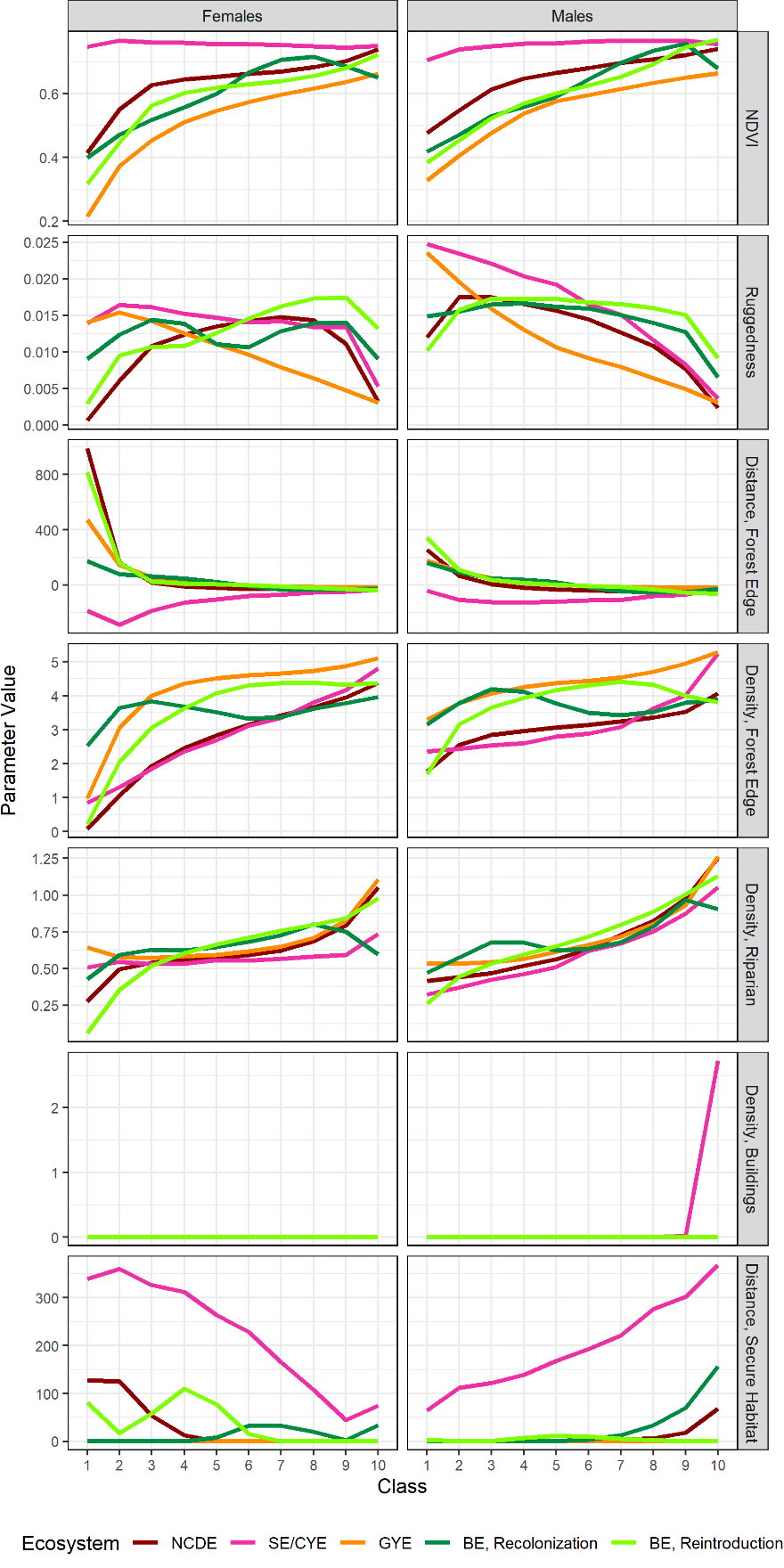


**A12.** Mean values of habitat variables used by grizzly bears, as measured within each of the 10 quantiles of predicted habitat use within the NCDE, SE/CYE, and GYE, and within the BE study area based on our natural recolonization and reintroduction simulations.


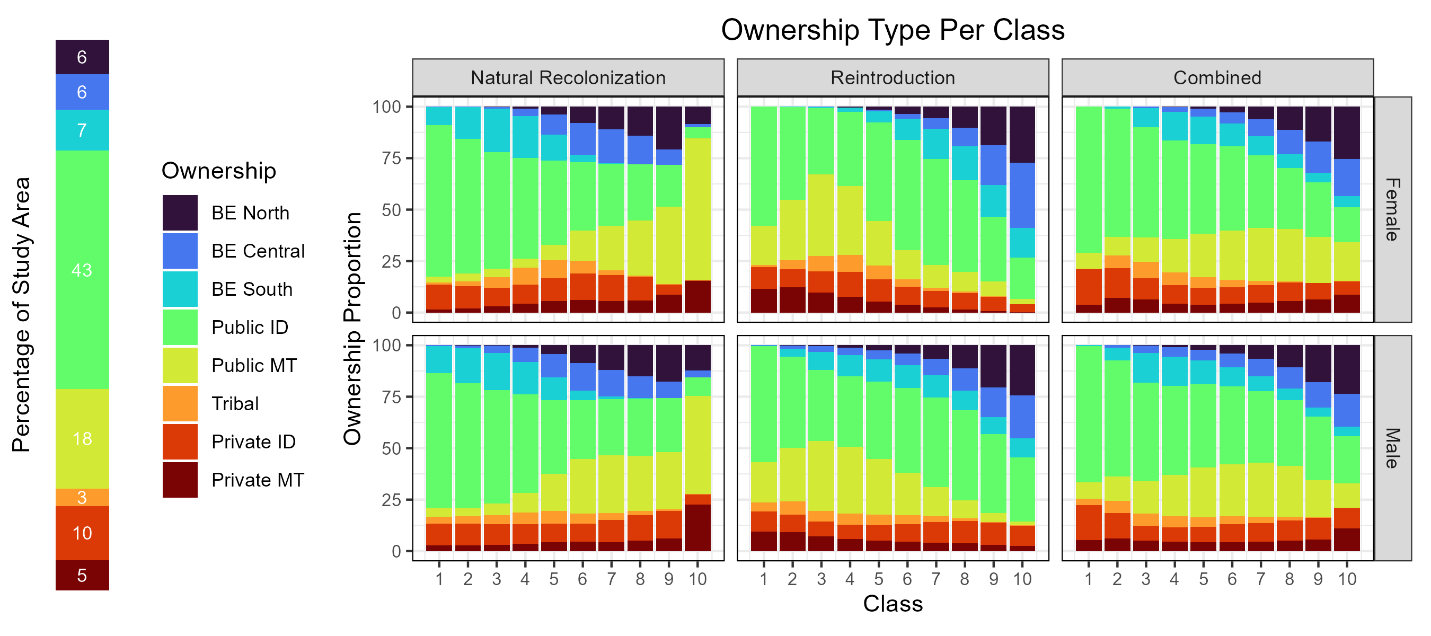


**A13.** The proportion of ownership or jurisdiction type in the study area (left panel) and the contribution of each type to the classes of habitat use (1 = lowest, 10 = highest) by simulated female (top) and male (bottom) bears, under natural recolonization, reintroduction, or combined scenarios.

**References**

1. Sells SN, Costello CM, Lukacs PM, Roberts LL, Vinks MA. Grizzly bear habitat selection across the Northern Continental Divide Ecosystem. Biological Conservation. 2022; 276.

2. Avgar T, Potts JR, Lewis MA, Boyce MS, Börger L. Integrated step selection analysis: bridging the gap between resource selection and animal movement. Methods in Ecology and Evolution. 2016; 7(5):619-30.

3. Signer J, Fieberg J, Avgar T. Animal movement tools (amt): R package for managing tracking data and conducting habitat selection analyses. Ecol Evol. 2019; 9(2):880-90.

4. Stoneberg RP, Jonkel CJ. Age determination of black bears by cementum layers. Journal of Wildlife Management. 1966; 30(2):411-4.

5. R. Core Team. R: A language and environment for statistical computing. Vienna, Austria: R Foundation for Statistical Computing; 2020.

6. Busetto L, Ranghetti L. MODIStsp: an R package for preprocessing of MODIS Land Products time series. Computers and Geosciences. 2016; 97:40-8.

7. Peck CP, VanManen FT, Costello CM, Haroldson MA, Landenburger LA, Roberts LL, et al. Potential paths for male-mediated gene flow to and from an isolated grizzly bear population. Ecosphere. 2017; 8(10).

8. Hollister J, Shah T, Nowosad J, Robitaille AL, Beck MW, Johnson M. elevatr: Access Elevation Data from Various APIs. 2023.

9. Evans JS. spatialEco. 2018. https://github.com/jeffreyevans/spatialEco.

10. Sappington JM, Longshore KM, Thomson DB. Quantifying landscape ruggedness for animal habitat analysis: a case study using bighorn sheep in the Mojave Desert. Journal of Wildlife Management. 2007; 71(5):1419-26.

11. Hijmans RJ. raster: Geographic data analysis and modeling. 2022.

12. Schwartz CC, Haroldson MA, White GC. Hazards affecting grizzly bear survival in the Greater Yellowstone Ecosystem. Journal of Wildlife Management. 2010; 74(4):654-67.

13. Juliusson L, Fortin-Noreus JK. Grizzly Bear Secure Core Habitat Analysis for the San Juan and Sierra Nevada Mountains’ Historical Range. US Fish and Wildlife Service; 2020.

14. Boyce MS, Vernier PR, Nielsen SE, Schmiegelow FKA. Evaluating resource selection functions. Ecological Modelling. 2002; 157:281-300.

15. Carnahan AM, Manen FTV, Haroldson MA, Stenhouse GB, Robbins CT. Quantifying energetic costs and defining energy landscapes experienced by grizzly bears. Journal of Experimental Biology. 2021; 224(6):1-9.

16. Schwartz CC, Miller SD, Haroldson MA. Grizzly Bear. 2003. p. 556-86.

17. US Fish and Wildlife Service. Grizzly Bear in the Lower 48 States 5-Year Status Review: Summary and Evaluation. Denver, Colorado; 2021.

18. Sells SN, Costello CM, Lukacs PM, Roberts LL, Vinks MA. Predicted connectivity pathways between grizzly bear ecosystems in Western Montana. Biological Conservation. 2023; 284.

**Note:** Any use of trade, firm, or product names is for descriptive purposes only and does not imply endorsement by the U.S. Government.
